# Human Astrocytes Transfer Aggregated Alpha-Synuclein via Tunneling Nanotubes

**DOI:** 10.1523/JNEUROSCI.0983-17.2017

**Published:** 2017-12-06

**Authors:** Jinar Rostami, Staffan Holmqvist, Veronica Lindström, Jessica Sigvardson, Gunilla T Westermark, Martin Ingelsson, Joakim Bergström, Laurent Roybon, Anna Erlandsson

**Affiliations:** ^1^Molecular Geriatrics, Department of Public Health and Caring Sciences, Rudbeck Laboratory, Uppsala University 75185 Uppsala, Sweden,; ^2^Department of Medical Cell Biology, BMC, Uppsala University, 751 23 Uppsala, Sweden,; ^3^Stem Cell Laboratory for CNS Disease Modeling, Wallenberg Neuroscience Center, Department of Experimental Medical Science,; ^4^Strategic Research Area MultiPark, and; ^5^Lund Stem Cell Center, Lund University, 22184 Lund, Sweden, and; ^6^BioArctic AB, 112 51 Stockholm, Sweden

**Keywords:** alpha-synuclein, astrocytes, lysosomes, mitochondria, trans-Golgi, tunneling nanotubes

## Abstract

Many lines of evidence suggest that the Parkinson's disease (PD)-related protein α-synuclein (α-SYN) can propagate from cell to cell in a prion-like manner. However, the cellular mechanisms behind the spreading remain elusive. Here, we show that human astrocytes derived from embryonic stem cells actively transfer aggregated α-SYN to nearby astrocytes via direct contact and tunneling nanotubes (TNTs). Failure in the astrocytes' lysosomal digestion of excess α-SYN oligomers results in α-SYN deposits in the trans-Golgi network followed by endoplasmic reticulum swelling and mitochondrial disturbances. The stressed astrocytes respond by conspicuously sending out TNTs, enabling intercellular transfer of α-SYN to healthy astrocytes, which in return deliver mitochondria, indicating a TNT-mediated rescue mechanism. Using a pharmacological approach to inhibit TNT formation, we abolished the transfer of both α-SYN and mitochondria. Together, our results highlight the role of astrocytes in α-SYN cell-to-cell transfer, identifying possible pathophysiological events in the PD brain that could be of therapeutic relevance.

**SIGNIFICANCE STATEMENT** Astrocytes are the major cell type in the brain, yet their role in Parkinson's disease progression remains elusive. Here, we show that human astrocytes actively transfer aggregated α-synuclein (α-SYN) to healthy astrocytes via direct contact and tunneling nanotubes (TNTs), rather than degrade it. The astrocytes engulf large amounts of oligomeric α-SYN that are subsequently stored in the trans-Golgi network region. The accumulation of α-SYN in the astrocytes affects their lysosomal machinery and induces mitochondrial damage. The stressed astrocytes respond by sending out TNTs, enabling intercellular transfer of α-SYN to healthy astrocytes. Our findings highlight an unexpected role of astrocytes in the propagation of α-SYN pathology via TNTs, revealing astrocytes as a potential target for therapeutic intervention.

## Introduction

Cellular inclusions in the brain, often referred to as Lewy bodies and Lewy neurites, are a pathological hallmark for Parkinson's disease (PD) and several other neurodegenerative disorders, including dementia with Lewy bodies and multiple system atrophy (Spillantini et al., 1998). The inclusions predominantly consist of insoluble fibrillary forms of the α-synuclein (α-SYN) protein (Spillantini et al., 1997), but smaller soluble aggregates are also present in the diseased brain. These aggregates, referred to as α-SYN oligomers, are particularly neurotoxic (Danzer et al., 2007; Chinta et al., 2010; Winner et al., 2011; Luth et al., 2014; Ogen-Shtern et al., 2016). Although α-SYN deposits are primarily found in neurons, they also appear frequently in glial cells at advanced disease stages (Tu et al., 1998; Wakabayashi et al., 2000; Terada et al., 2003; Croisier and Graeber, 2006; Braak et al., 2007). However, the consequences of astrocytic α-SYN inclusions for progression and spreading of PD pathology remain unknown.

Being the most abundant glial cell type in the nervous system, astrocytes play an important role in maintaining brain homeostasis (Sofroniew and Vinters, 2010). The complex role of astrocytes in the pathological brain is largely dependent on their release and uptake of substances from the microenvironment that they share with the neurons (Sofroniew and Vinters, 2010). For example, astrocytes confer neuroprotection by removing excessive extracellular glutamate, potassium, and calcium, whereas they produce cytokines and chemokines that could be harmful to neurons if released chronically (Rappold and Tieu, 2010; Sofroniew and Vinters, 2010).

Reactive astrocytes effectively engulf dead cells, synapses, and protein aggregates of amyloid β (Aβ) and α-SYN (Chang et al., 2000; Magnus et al., 2002; Sokolowski et al., 2011; Lööv et al., 2012; Chung et al., 2013; Fellner et al., 2013; Jones et al., 2013; Söllvander et al., 2016). Moreover, experimental evidence from *in vitro* and *in vivo* studies indicates that α-SYN can transfer from cell to cell and thereby contribute to disease progression (Kordower et al., 2008; Li et al., 2008; Desplats et al., 2009; Lee et al., 2010; Danzer et al., 2011; Hansen et al., 2011). Several transfer mechanisms have been suggested, including exocytosis/endocytosis, extracellular vesicle secretion, and prion-like spreading, but many questions remain regarding α-SYN spreading and the involvement of various cell types, notably astrocytes (Gousset et al., 2009; Jang et al., 2010; Emmanouilidou et al., 2011).

Tunneling nanotubes (TNTs) are thin protrusions that allow direct physical connections of the plasma membranes between remote cells. The existence of TNTs was described for the first time in 2004 (Rustom et al., 2004). Subsequently, TNTs have been found in numerous cell types, including neurons and glial cells (Zhu et al., 2005; Wang et al., 2012). Their primary function is to transfer cellular components such as organelles, proteins, genetic materials, ions, and small molecules between cells over long distances (Kimura et al., 2013). However, TNTs have also been suggested to be important in pathological conditions by facilitating the intercellular spreading of viruses and pathogenic proteins (Rustom et al., 2004). The smallest TNTs (<100 nm in diameter) only contain F-actin, whereas thicker TNTs (>100 nm in diameter) are composed of both F-actin and microtubules and allow cell-to-cell transfer of larger compartments such as organelles (Wang et al., 2011; Sisakhtnezhad and Khosravi, 2015). Therefore, TNT formation can effectively be inhibited by F-actin depolymerization drugs such as latrunculin B (Bukoreshtliev et al., 2009).

Knowledge about the cellular mechanisms behind the initiation and propagation of PD is still very limited. Here, we show that human astrocytes engulf large amounts of α-SYN oligomers that are subsequently stored in the trans-Golgi region. The accumulation of α-SYN in the astrocytes affects their phagosomal–lysosomal machinery and induces mitochondrial damage. As a consequence of these stress reactions, the α-SYN-containing astrocytes protrude TNTs, which mediate transfer of α-SYN aggregates and mitochondria between neighboring cells. Together, our results highlight an unexpected role of astrocytes in the progression of α-SYN pathology.

## Materials and Methods

### 

#### α-SYN oligomer generation and labeling

**Figure 1.**
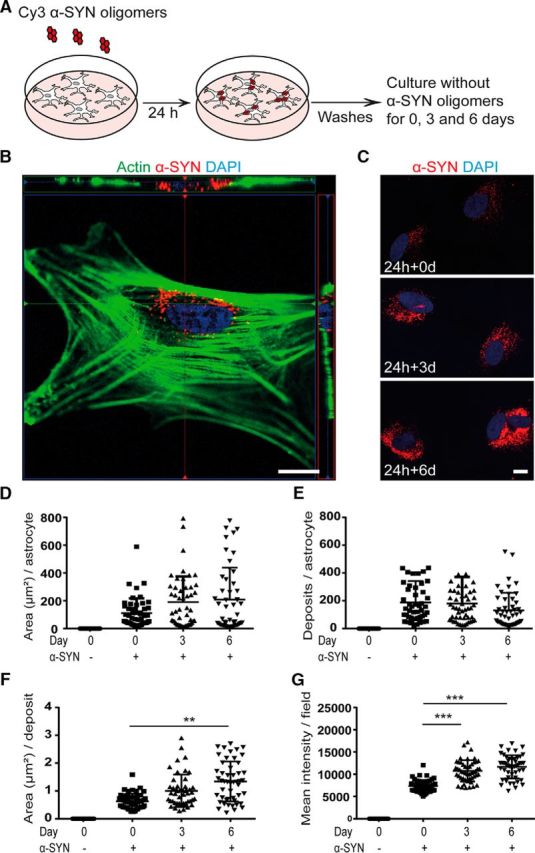
Intracellular α-SYN is stored rather than degraded. Human ESC-derived astrocytes (see Fig. 1-1*A*) were exposed to 0.5 μm Cy3-labeled α-SYN oligomers (see Fig. 1-1*B*) for 24 h, washed thoroughly, and cultured for an additional 0, 3, or 6 d in α-SYN-free medium before fixation (***A***). The intracellular location of the α-SYN-Cy3 after ingestion (see Fig. 1-2*A*) was confirmed with confocal imaging (***B***). Quantification of the α-SYN-Cy3 signal at days 0, 3, and 6 (***C*** and Fig. 1-2*B*) reveled the total area of the α-SYN deposits per astrocyte in square millimeters (***D***), the number of α-SYN deposits per astrocyte (***E***), the mean area of the deposits in square millimeters (***F***), and the mean intensity of the intracellular α-SYN-Cy3 per field (***G***). Western blot analysis confirmed the presence of high-molecular-weight α-SYN species in the astrocytes at all three time points (Fig. 1-2*C*), demonstrating that only minimal degradation of the ingested oligomeric α-SYN occurred, whereas the monomeric α-SYN was completely degraded during the 6 d time period (see Fig. 1-3*A*, *B*). Scale bars: ***B***, 10 μm; ***C***, 20 μm. Data are presented as mean ± SD from four independent experiments and the levels of significance were set to *p < 0.05, **p < 0.01, and ***p < 0.001 (***E***–***G***).

Recombinant α-SYN (140 μm) was produced as described previously (Näsström et al., 2011). Monomeric α-SYN was incubated with 4-hydroxy-nonenal (HNE) (Cayman Chemicals) in a HNE: α-SYN ratio of 30:1 at 37°C for 72 h. SEC-HPLC was performed on a Superose 6 PC 3.2/30 column (GE Healthcare) and revealed a near complete conversion from monomers to oligomers (Fig. 1-1*B*). As an eluent, 20 mm Tris and 0.15 m NaCl, pH 7.4, was used at a flow rate of 50 μl/min. The α-SYN oligomers were labeled with the Cy3^AM^ Antibody Labeling Kit (GE Healthcare, PA33000), the Atto 488 protein labeling kit (Sigma-Aldrich) or pHrodo (Invitrogen, P36600). The pHrodo dye reacts with amine groups on the cell surface and is nonfluorescent at neutral pH, but emits red light at an increasing intensity as the pH is lowered. For all labeling, the protocols provided with the dye kits were followed and unbound excess Cy3, Atto 488, or pHrodo were removed by filtration in a Zeba spin desalting column (Thermo Scientific) according to the manufacturer's instructions.

#### Culture of human embryonic stem cell (ESC)-derived astrocytes

Human ESC-derived astrocytes were generated as described previously (Holmqvist et al., 2015; Stem Cell Research). The cells were cultured in Advanced DMEM/F12 (Thermo Fisher Scientific, 12634-010) supplied with 1% FBS (Thermo Fisher Scientific, 10082-147), 1% penicillin/streptavidin (Thermo Fisher Scientific, 15140-122), 2 μg/ml heparin (Sigma-Aldrich, H4784), 10% B27 supplement (Thermo Fisher Scientific, 17504-044), 1% nonessential amino acids (Merck Millipore, TMS001-C), and 1% l-glutamine (Thermo Fisher Scientific, 25030-024). Cells were passaged using Trypsin-EDTA (Life Technologies). Cells from 90 to 120 d *in vitro* (DIV) were used in the study. For experiments, the astrocytes were seeded at a concentration of 1500 cells/cm^2^, resulting in a 20–30% confluence. TUNEL analysis showed that apoptotic cell death in the culture was <3% (Fig. 3-1*B*,*C*).

#### Alpha-synuclein exposure

Astrocyte cultures were exposed to 0.5 μm α-SYN oligomers or α-SYN monomers for 24 h. This concentration was chosen based on our previous investigations of neuronal–glial cocultures, demonstrating that astrocytes rapidly internalized particularly large amounts of α-SYN oligomers (Lindström et al., 2017). After exposure, the cells were thoroughly washed in medium two times and continuously cultured in medium without oligomers or monomers. At 0, 3, and 6 d after exposure, the cells were fixed for further analyses. Parallel control cultures were left untreated and fixed at the same time points.

#### Time-lapse microscopy

Cells were recorded using time-lapse microscopy (Nikon Biostation IM Cell Recorder). Images were taken at 20×, 40×, or 80 × magnifications every 5 or 10 min. The duration of the experiment was 24 h during the α-SYN oligomer exposure or 72 h after the 24 h α-SYN oligomer exposure (and wash).

#### Western blot analysis

At 0, 3, and 6 d after α-SYN oligomer and monomer exposure, cells were lysed in lysis buffer containing 20 mm Tris, pH 7.5, 0.5% Triton X-100, 0.5% deoxycholic acid, 150 mm NaCl, 10 mm EDTA, 30 mm NaPyroP, 500 μm sodium orthovanadate, and 1× protease inhibitor (Thermo Fisher Scientific, 78430) on ice for 30 min before centrifugation for 30 min at 4°C 12,000 × *g*. The supernatants were transferred to new tubes and the pellets and lysates were kept in −70°C until analysis. To study autophagic flux, control cells and α-SYN oligomer-exposed cells were treated with bafilomycin (500 nm, Millipore) at 24 h + 6 d for 6 h before lysis. Protein concentration was measured using the BCA protein assay kit (Thermo Fisher Scientific, 23225) according to the manufacturer's protocol.

A total volume of 40 μl of each sample, containing 20 μg of protein, 1× Bolt Sample Reducing agent (Thermo Fisher Scientific), and 1× NuPAGE LDS sample buffer (Thermo Fisher Scientific), was loaded to a 4–12% NuPAGE Bis-Tris gel (Thermo Fisher Scientific). Chameleon kit 700 and 800 prestandard protein ladders (LICOR) were mixed and added to the gel. The gel was run at 200 V for 30 min in Bolt MES SDS Running buffer (1×, Thermo Fisher Scientific). The PVDF membrane was activated in methanol for 5 min before transfer at 22 V for 1 h in MES transfer buffer (1×) supplied with 10% methanol, 0,1% Bolt antioxidants (Thermo Fisher Scientific) and 0.01% SDS. For α-SYN monomer and oligomer detection, membranes were fixed with 0.4% PFA for 30 min and washed with PBS 3× 5 min before blocking in 5% BSA in TBS for 1 h at room temperature. The membranes were then incubated with primary antibodies overnight at 4°C. The primary antibodies used were as follows: LAMP-1 (1:1000, Abcam, ab24170), LC3B (1:500, Novus Biologicals, NB100-2220SS), p62 (1:500, Novus Biologicals, NBP1-48320SS), GAPDH (Novus Biologicals, NB300-221), and FL-140 (1:1000, Santa Cruz Biotechnology). The following day, the membranes were washed in 0,1% Tween in TBS (TBS-T) 3× 10 min before incubation with HRP-coupled secondary antibody (1:20,000, Pierce) for 1 h at room temperature. After 3× 10 min washes with TBS-T, the membranes were incubated with the ECL prime Western blotting detection reagents (1:1 mixture of reagent A and B, GE Healthcare) and the signal was measured using the ChemiDoc XRS machine (Bio-Rad).

To detect α-SYN oligomers in the pellet, samples were resuspended in 1% SDS and 1× protease inhibitor in TBS. The samples were boiled in 95°C for 5 min before sonication for 30 s with 1 s pulse on/off and an amplitude of 20%. The samples were treated as the other samples before loading to the gel.

#### Luminescent ATP detection kit

Cells were grown in 24-well plates (5,000 cells/cm^2^ cells per well) and exposed to α-SYN oligomers as described previously. The total ATP levels were analyzed at 24 h + 6 d with the Luciferase-Based Luminescent ATP Detection Assay Kit (Abcam, ab113849) according to the manufacturer's protocol. As a positive control, cells were incubated with a 5 μm concentration of the mitochondrial electron transport inhibitor antimycin A (Sigma-Aldrich, A8674) for 21 h before measurement. Luminescence was measured with an Infinite M1000 plate reader (Tecan). The assay was performed in a biological replicate of *n* = 6; that is, from 6 different batches of cells. Luminescence values are presented as the relative change compared with untreated control cultures.

#### Coculture experiments with RFP/GFAP-expressing astrocytes

Different coculture systems were used to study cell-to-cell transfer of α-SYN oligomers and mitochondria. Unlabeled astrocytes and astrocytes expressing tagged-RFP (tRFP) under the GFAP_ABC1D_ promoter (Holmqvist et al., 2015) were used as either acceptor or donor cells. The different combinations are described below.

##### α-SYN transfer to unexposed, tRFP acceptor astrocytes.

Unlabeled astrocytes were treated with Atto 488-labeled α-SYN oligomers for 24 h followed by washes. At 24 h + 2 d, the exposed astrocytes (donor cells) were trypsinized and added to the unexposed tRFP astrocytes (acceptor cells). After 24 h of coculture, the cells were fixed. Actin polymerization was inhibited by addition of latrunculin B (1 μm, Sigma-Aldrich) 3 h after the coculture was started.

##### Mitochondria transfer from α-SYN oligomer-exposed astrocytes to unexposed, tRFP acceptor astrocytes.

Unlabeled astrocytes were treated with unlabeled α-SYN oligomers for 24 h followed by washes. At 24 h + 1 d, the α-SYN oligomer-exposed astrocytes were transfected with cellLight mitochondria-GFP (Mitotracker, Thermo Fisher Scientific) at 20 particles per cell (PPC) (donor cells). Unexposed, tRFP astrocytes (acceptor cells) were added to the donor cells at 24 h + 2 d. After 24 h of coculture, the cells were fixed. Actin polymerization was inhibited using latrunculin B (1 μm) 3 h after the coculture was started.

##### Mitochondria transfer from unexposed tRFP astrocytes to α-SYN oligomer-exposed acceptor astrocytes.

Unlabeled astrocytes were treated with unlabeled α-SYN oligomers for 24 h followed by washes. At 24 h + 1 d unexposed, tRFP astrocytes were transfected with Miotracker (20 PPC, donor cells). The tRFP astrocytes (donor cells) were added to the α-SYN oligomer-exposed astrocytes at 24 h + 2 d (acceptor cells), followed by 24 h of coculture and fixation. Actin polymerization was inhibited using latrunculin B (1 μm) 3 h after the coculture was started. Mitochondrial transfer was also studied in parallel cocultures in which neither the acceptor cells nor the donor cells were treated with α-SYN oligomers.

#### Immunocytochemistry

Cells were fixed in 4% PFA in PBS, washed, and blocked with 5% normal goat serum (NGS) and 0.1% Triton in PBS for 30 min in room temperature. Primary antibodies were diluted in 0.5% NGS and 0.1% Triton in PBS and added to the cells for 2 h in room temperature. Thereafter, cells were washed 3× with PBS before incubation with secondary antibodies and dyes for 45 min at 37°C. After additional washes, cells were mounted with Vectashield Hard Set Mounting medium with DAPI or without DAPI (BioNordika) and analyzed using the fluorescence microscope Observer Z1 Zeiss. Confocal images were taken using Zeiss LSM700 and LSM 710 and 3D images were performed using the IMARIS 8.2 program. The following primary antibodies were used: anti-nestin (1:400, Millipore, ABD69), anti-S100B (1:200, Sigma-Aldrich, S2532), anti-vimentin (1:200, Abcam, ab5733), anti-LAMP1 (1:200, Abcam, ab24170), anti-TGN46 (1:100, Abcam, ab50595), anti-COXIV (mouse monoclonal, 1:100, Abcam, ab14744), anti-COXIV (rabbit polyclonal, 1:100, Abcam, ab16056), anti-DRP-1 (1:200, Abcam, ab56788), anti-LC3B (1:200, Abcam, ab51520), anti-Golgi complex antibody (1:200, Abcam, ab103439), and anti-calnexin (1:200, Santa Cruz Biotechnology, sc11397). The following secondary antibodies and dyes were used: Alexa Fluor 488 goat anti rabbit/mouse (1:200, Abcam), Alexa Fluor Cy3 goat anti rabbit/mouse (1:200, Abcam), and Alexa Fluor 488 phalloidin (Sigma-Aldrich). To stain the plasma membrane, fixed cells were washed 3× with HBSS (Life Technologies) before incubation with wheat germ agglutinin (WGA), Alexa Fluor 350 conjugate (1:200, Life Technologies) for 10 min. Astrocytic apoptosis was measured using terminal (TdT)-mediated dUTP-biotin reaction mixture (TUNEL, Roche Biochemicals) according to the manufacturer's instructions. Mitochondria were labeled with cellLight mitochondria-GFP BacMam 2.0 (Thermo Fisher Scientific) and LC3B^+^ vesicles were labeled with the Premo Autophagy Tandem Sensor RFP-GFP-LC3B Kit. As a control to the Autophagy Tandem Sensor RFP-GFP-LC3B Kit, 90 μm chlouroscin was added to the cells for 8 h before fixation. The transfection reagents were mixed in the cell medium at 20 PPC and added to the cells for 24 h before fixation.

#### Transmission electron microscopy (TEM)

Astrocyte cultures were fixed in 2.5% glutaraldehyde in 0.1 m sodium cacodylate buffer (SCB), pH 7.4. The cell culture dishes were then rinsed in 0.1 m SCB for 10 min and incubated in 1% OsO_4_ in 0.1 m SCB for 1 h. Dehydration was performed with 70% ethanol for 30 min, 95% ethanol for 30 min, and 99.7% ethanol for 1 h. The dishes were rinsed with plastic (Agar 100 resin kit, Agar Scientific) and a new, thin layer of plastic was added to the cells for 2–4 h to permit evaporation of the alcohol. A second plastic layer was poured on and left overnight before a thicker, newly made plastic layer was added. The dishes were incubated at room temperature for 1 h before polymerization in the oven (60°C) for 48 h. The cells were studied in a Hitachi H-7100 TEM.

#### Quantifications and statistics

##### Intensity measurements of α-SYN deposits.

In total, 12 images per experiment and time point were captured at days 0, 3, and 6 after α-SYN oligomer exposure. Zen 2012 software was used to analyze the intensity and number of α-SYN inclusions from four independent experiments. The data were analyzed in GraphPad Prism 6.0 software using Kruskal–Wallis statistical analysis.

##### Analysis of TUNEL assay.

In total, 12 images per experiment and time point were captured at days 0, 3, and 6 after α-SYN oligomer exposure or latrunculin B treatment and in parallel control cultures. Two independent experiments were performed and the number of TUNEL^+^ cells and the total cell number were determined manually. The data were analyzed in GraphPad Prism 6.0 using two-way ANOVA statistical analysis.

##### Intensity measurement of Western blot analysis.

The LC3BII/I ratio, normalized to GAPDH, and the p62 levels, normalized to GAPDH, were measured in three independent experiments using Image Lab software. The data were analyzed in GraphPad Prism 6.0 using one-way ANOVA statistical analysis.

##### Analysis of autophagic flux using LC3B tandem.

To determine whether the autolysosomes were affected by the α-SYN treatment, astrocytes were transfected with LC3B tandem where the LC3B protein is coupled to RFP and pH-sensitive GFP. When the autophagosomes fuse with the lysosomes, the pH-sensitive GFP will be degraded. Twenty cells from each time point were analyzed. Cells with only RFP^+^/GFP^+^ vesicles and cells with a mixture of RFP^+^ and RFP^+^/GFP^+^ vesicles were counted manually in four independent experiments and analyzed using one-way ANOVA statistical analysis.

##### Morphology analysis of endoplasmic reticulum (ER) in TEM images.

Images were captured in close proximity to the nucleus with a magnification of 20,500× and the ER width was measured using ImageJ software. In total, the ER width from 14 α-SYN oligomer-exposed astrocytes and 13 untreated, control astrocytes were measured. The data were analyzed in GraphPad Prism 6.0 using Student's *t* test statistical analysis.

##### Morphology analysis of mitochondria in TEM images.

For measurements of the effect of α-SYN oligomers on mitochondrial fusion/fission, the mitochondria were divided into two groups depending on their length: normal mitochondria (>1 μm) and dense/fragmented mitochondria (<1 μm). Mitochondria within the two groups were quantified in 14 α-SYN oligomer-exposed astrocytes and in 12 untreated, control astrocytes. The data were analyzed in GraphPad Prism 6.0 using two-way ANOVA statistical analysis.

##### Morphology analysis of mitochondria in immunocytochemistry images.

COXIV^+^ mitochondrial clumps were measured using ImageJ software in 20 images per experiment and time point at days 0, 3, and 6 after α-SYN-Cy3 exposure and in parallel control cultures. Three independent experiments were performed and the data were analyzed in GraphPad Prism 6.0 using Kruskal–Wallis statistical analysis.

##### Quantification of TNTs.

For analysis, TNTs were stained with phalloidin and WGA. The inclusion criterion for TNTs herein was protrusions connecting two cells with a length of 5–100 μm. In total, 20 images per experiment and time point were captured at day 0, 3, and 6 cultures after α-SYN oligomer exposure and in parallel control cultures. Four independent experiments were performed and the number of TNTs was determined manually and analyzed using ANOVA statistical analysis. Actin polymerization was inhibited by the addition of latrunculin B 2 d after the 24 h α-SYN oligomer exposure. The cell cultures were fixed 1 d later (at day 3 after the α-SYN oligomer exposure). The effect of latrunculin B on TNT formation was analyzed in 20 images per experiment in α-SYN oligomer-exposed astrocytes in the absence and presence of latrunculin B. Four independent experiments were performed and the data were analyzed using Student's *t* test.

##### Quantifications of transfer in the cocultures.

Mitochondria and α-SYN^+^ acceptor cells in the absence and presence of latrunculin B were counted in 16 images per experiment. Three independent experiments were performed and the data were analyzed using Mann–Whitney statistical analysis.

## Results

### α-SYN oligomers accumulate in astrocytes after ingestion

**Figure 2.**
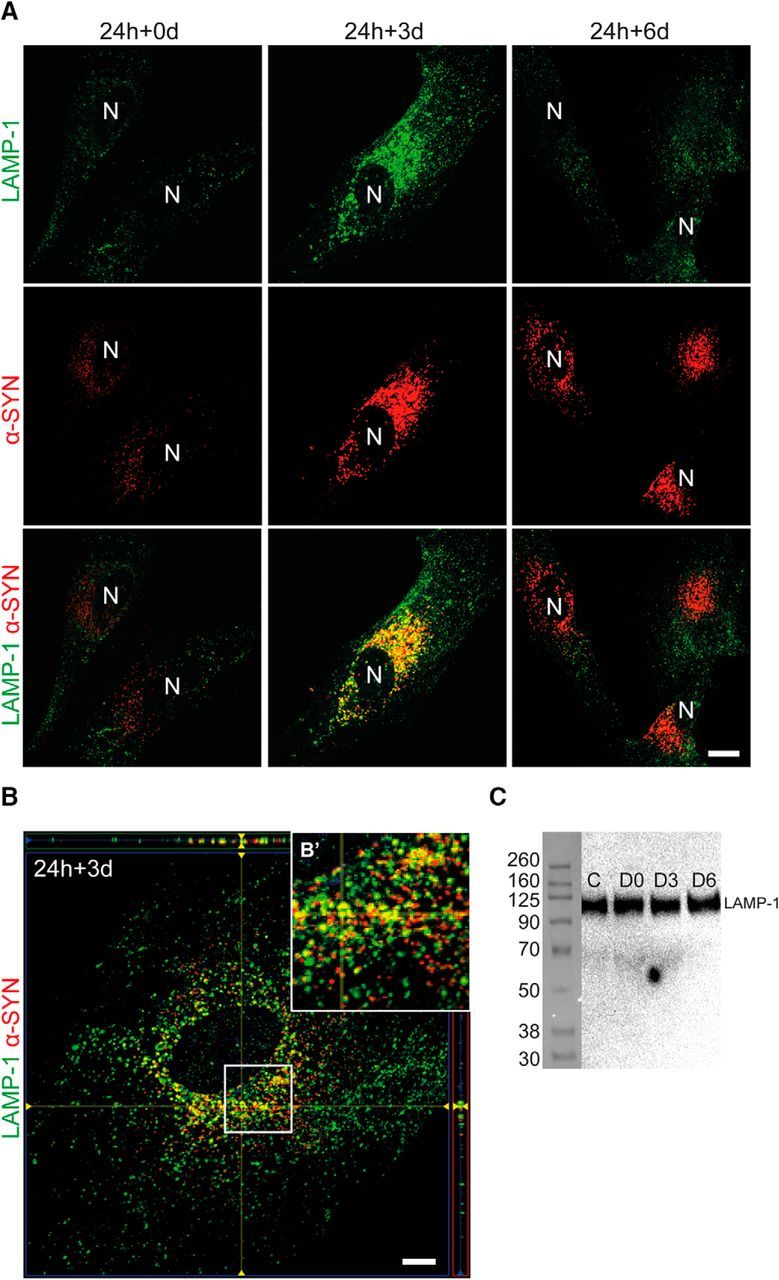
Ingested α-SYN only temporarily colocalizes with the lysosomal protein LAMP-1. Representative images of LAMP-1 staining from α-SYN oligomer-exposed astrocytes and control cells showed a pronounced colocalization of LAMP-1 and α-SYN at day 3 (24 h + 3 d). At day 6 (24 h + 6 d), the colocalization was lost, although the α-SYN deposits were not degraded (***A***, Fig. 2-1, and Fig. 2-2). N, Nucleus. Confocal imaging demonstrated clear colocalization of LAMP-1 and α-SYN at day 3 (24 h + 3 d) (***B***). A close-up of the white rectangle is shown in ***B′***. Western blot analysis revealed no differences in LAMP-1 protein expression between the control and the α-SYN oligomer-exposed astrocytes (***C***). Scale bars: ***A***, 20 μm; ***B***, 10 μm.

**Figure 3.**
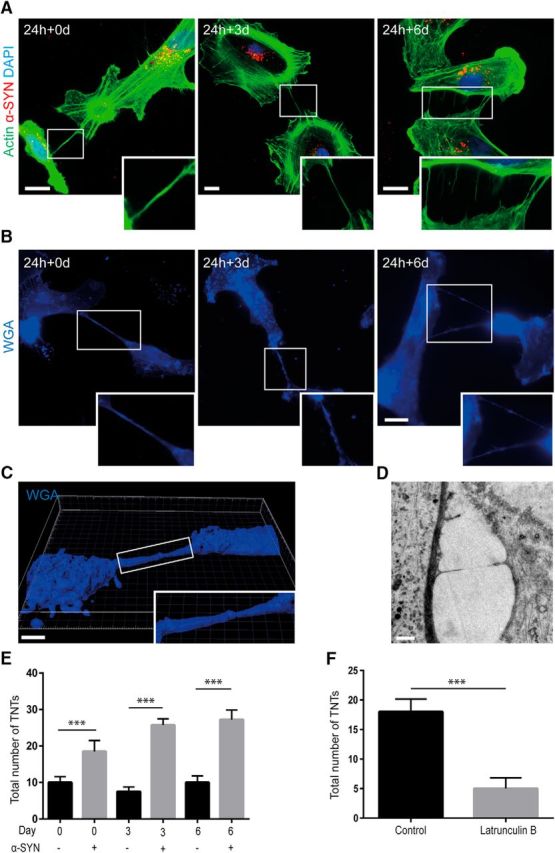
Accumulation of α-SYN oligomers induces TNT formation. F-actin labeling using phalloidin (***A***) and membrane staining using WGA (***B***) showed that TNTs were formed between the astrocytes at 0, 3, and 6 d (24 h + 0 d, 24 h + 3 d, and 24 h + 6 d) after α-SYN oligomer exposure. Confocal 3D imaging (***C*** and Fig. 3-1*A*) and TEM analysis (***D***) confirmed the presence of TNTs. Quantification of the total number of TNTs in cultures treated with α-SYN oligomers or in parallel control cultures showed that the number of TNTs was increased significantly after α-SYN oligomer exposure at all time points. Moreover, the number of TNTs increased over time in the α-SYN oligomer-treated cultures (***E***). Latruculin B treatment reduced the number of TNTs significantly (***F***). Importantly, neither the high load of α-SYN oligomers nor the treatment with latrunculin B at the concentration and exposure time used induced any obvious toxicity or cell death, as indicated by TUNEL staining and the total cell number (Fig. 3-1*B*, *C*). Scale bars: ***A***, ***B***, 20 μm; ***C***, 5 μm; ***D***, 1 μm. Data are presented as mean ± SD from four independent experiments and the levels of significance were set to *p < 0.05, **p < 0.01, and ***p < 0.001 (***E***, ***F***).

Human ESC-derived astrocytes (Holmqvist et al., 2015) expressing the markers nestin, vimentin, S100β, and GFAP (Fig. 1-1*A*) were exposed to, 0.5 μm Cy3-labeled α-SYN oligomers (Fig. 1-1*B*) for 24 h (see schematic outline in Fig. 1*A*). Compared with S100β and nestin, the expression of GFAP was rather low, but none of the astrocytes were completely GFAP^−^. Live-cell imaging starting instantly after exposure visualized uptake of α-SYN oligomers (starred in the figures) in the astrocytes (Fig. 1-2*A*). After engulfment (Fig. 1-2*A*, 11 h 25 min to 11 h 55 min), the astrocytes contracted and temporarily rounded up as α-SYN was transported to the region around the nuclei (Fig. 1-2*A*, 19 h 05 min to 21 h 36 min), where it persisted throughout the time-lapse experiment. Contraction after engulfment of large pathogens have been described previously in other phagocytic cells (Evans et al., 1993). Moreover, we confirmed the intracellular location of the engulfed α-SYN-Cy3 oligomers in the astrocytes by actin labeling and confocal 3D imaging (Fig. 1*B*). To investigate whether the α-SYN inclusions were degraded by the human astrocytes, the cells were thoroughly washed after the 24 h α-SYN-Cy3 oligomer exposure and cultured for an additional 3 or 6 d in α-SYN-free medium before fixation (see schematic outline in Fig. 1*A*). Interestingly, we found that the ingested α-SYN oligomers remained in the cells throughout the experiment (Fig. 1-2*B*). To verify this result, we performed Western blot analysis of total whole-cell lysates and pellet fractions with antibodies directed to α-SYN (Fig. 1-2*C*). The reason that we included analysis of the pellet fractions was that we have noted previously that intracellular deposits of amyloid-β in astrocytes end up in the pellet fraction due to their compact and large structure (Söllvander et al., 2016). As expected, SDS treatment of the α-SYN oligomers resulted in dissociation into a wide range of high- and low-molecular-weight α-SYN species (Fig. 1-2*C*; Näsström et al., 2011). Interestingly, our data show that there was a reduction in high-molecular-weight αSYN species in the lysates over time, but the high-molecular-weight αSYN species in the pellet fraction remained rather intact at all three time points, demonstrating that the α-SYN oligomers were not effectively degraded by the cells over the 6 d time period (Fig. 1-2*C*). The immunostainings showed that all astrocytes in the α-SYN-exposed cultures had α-SYN deposits and we did not observe any obvious difference in uptake or accumulation of α-SYN in cells with high versus low GFAP expression. In contrast to the oligomers, monomeric α-SYN was degraded rapidly by the human astrocytes (Fig. 1-3, *A*,*B*).

10.1523/JNEUROSCI.0983-17.2017.f1-1 Figure 1-1 Immunocytochemical characterization of the human ESC-derived astrocytes demonstrated positive staining using specific antibodies to vimentin, nestin, S100β and GFAP (A). SEC-HPLC chromatogram of the α-SYN oligomers and monomers demonstrates an almost complete conversion of monomers into oligomers (B). Scale bars = 20μm. Download Figure 1-1, TIF file

10.1523/JNEUROSCI.0983-17.2017.f1-2 Figure 1-2 Time lapse experiment demonstrated uptake of Cy3-α-SYN oligomers (yellow star) in astrocytes. Following engulfment (11h 25m to 11h 55 m), the astrocyte clearly contracted (white, dotted line indicates the border of the cell) and the α-SYN inclusions was transported towards the nuclei (N) (19h 05m to 21h 36 m) (A). Immunocytochemistry, demonstrated that ingested α-SYN aggregates were present intracellularly at 0, 3 and 6 days following the 24 h Cy3-α-SYN exposure (24h+0d, 24h+3d and 24h+6d) (B). Western blot analysis showed the presence of high molecular α-SYN species in the pellet fraction at all three time points (C). Scale bars (A) = 10 μm and (B) = 20μm. Download Figure 1-2, TIF file

10.1523/JNEUROSCI.0983-17.2017.f1-3 Figure 1-3 Monomeric Cy3-α-SYN was effectively degraded by the human ESC-derived astrocytes. At day 6 following α-SYN exposure (24h + 6 d) the Cy3-signal had disappeared completely (A). Western blot analysis confirmed that monomeric α-SYN was effectively cleared by the astrocytes (B). Scale bar = 20 μm. Download Figure 1-3, TIF file

Quantifications of the Cy3 signal at the different time points after oligomer exposure (Fig. 1*C*) demonstrated that the total area of the inclusions per astrocyte (Fig. 1*D*) and the number of deposits per astrocyte (Fig. 1*E*) were stable over time and did not change significantly from days 0 to 6, indicating minimal degradation of the ingested aggregated α-SYN during the 6 d time period. However, the mean area per inclusion (Fig. 1*F*) and the mean intensity per field (Fig. 1*G*) increased significantly over time (*p* < 0.001). These results could be explained by the fact that the aggregates were brought closer together over the 6 d period when the α-SYN was relocated to the region around the cell nuclei. This is a phenomenon the we demonstrated previously after astrocytic engulfment of amyloid-β oligomers (Söllvander et al., 2016).

### Lysosomal machinery fails to degrade ingested α-SYN oligomers

To determine whether the internalized α-SYN oligomers entered the lysosomal pathway, we performed immunostaining with endosomal/lysosomal-associated membrane protein 1 (LAMP-1). The stainings showed an increased perinuclear localization of LAMP-1 at day 3 after exposure to α-SYN oligomers compared with untreated control astrocytes (Fig. 2*A* and Fig. 2-1*A*,*B*). Moreover, we confirmed that, at this time point (24 h + 3 d), most of the intracellular α-SYN deposits colocalized with the LAMP-1^+^ vesicles (Fig. 2*A*,*B*). Notably, at day 6 (24 h + 6 d), the colocalization of LAMP-1 and α-SYN was reduced dramatically, although the α-SYN inclusions had not been degraded (Fig. 2*A* and Fig. 2-1). LAMP-1 staining was also less perinuclear at the latest time point without accomplishing the degradation (Fig. 2*A* and Fig. 2-1*A*). Western blot analysis of whole-cell lysates did not show any changes in total LAMP-1 expression between the control and α-SYN-exposed astrocytes (Fig. 2*C*), indicating that the endosomes/lysosomes were only redistributed inside the cells. Next, we investigated whether the engulfed α-SYN oligomers came into contact with mature lysosomes. For this purpose, α-SYN oligomers were prelabeled with the pH-dependent dye pHrodo before being added to the astrocytes. The pHrodo dye is only initiated to fluoresce at low pH such as that inside acidic lysosomes. We found pHrodo staining at all the time points studied (24 h + 0 d, 24 h + 3 d, and 24 h + 6 d), indicating that at least some of the ingested oligomers were transported to acidic lysosomes (Fig. 2-2). However, the pHrodo signal did not decline over time. Instead, the fraction of intracellular α-SYN that was pHrodo labeled accumulated further and formed deposits/inclusions (24 h + 6 d) in the perinuclear region, confirming severe failure of the lysosomal degradation. Therefore, the accumulation of pHrodo-labeled α-SYN was similar to the accumulation of Cy3-labeled α-SYN. These results show that the engulfed oligomeric α-SYN was transported to lysosomal compartments, but the astrocytes were incapable of degrading the α-SYN oligomers by the lysosomal machinery.

10.1523/JNEUROSCI.0983-17.2017.f2-1 Figure 2-1 Representative images from LAMP-1 and α-SYN immunostaining revealed co-localization between LAMP-1 and α-SYN at 24h+3d (A). Separate channels of the LAMP-1 staining of parallel untreated control cells at the different time points (B). Scale bars (A and B) = 20μm. Download Figure 2-1, TIF file

10.1523/JNEUROSCI.0983-17.2017.f2-2 Figure 2-2 Exposure of astrocytes to α-SYN oligomers pre-labeled with the pH-dependent dye pHrodo, demonstrated that although the engulfed oligomers were transported to acidic lysosomes, they were not effectively degraded. The pHrodo signal did not decline, instead the pHrodo positive α-SYN seemed to accumulate over time and formed larger inclusions at day 24h+6d. Scale bars = 20μm. Download Figure 2-2, TIF file

### Intracellular accumulation of α-SYN induces TNT formation

Cell-to-cell contacts via, for example, TNTs could facilitate intercellular spreading of pathogenic proteins and organelles. To investigate whether the high load of intracellular α-SYN deposits affected the formation of TNTs between the astrocytes in the culture, we stained the cellular cytoskeleton with the actin-binding dye phalloidin (Fig. 3*A*) and the plasma membrane with WGA (Fig. 3*B*). We found that TNTs were frequently formed between the astrocytes at 24 h + 0 d, 24 h + 3 d, and 24 h + 6 d (hereafter referred to as days 0, 3, and 6; Fig. 3*A*,*B*). Analysis with confocal microscopy (Fig. 3*C* and Fig. 3-1*A*) and TEM (Fig. 3*D*) further confirmed the presence of TNTs. Quantification of the total number of TNTs in cultures treated with α-SYN oligomers and in untreated control cultures showed that the α-SYN oligomer exposure clearly induced TNT formation because there were significantly more TNTs in the α-SYN oligomer-exposed cultures (Fig. 3*E*). Moreover, the number of TNTs increased over time (from 0–3 d and from 0–6 d, *p* < 0.001) after the 24 h of α-SYN oligomer exposure. Inhibition of actin polymerization by the addition of latrunculin B reduced the number of TNTs in the astrocyte culture significantly (Fig. 3*F*, *p* < 0.001). Importantly, neither the high load of α-SYN oligomers nor the treatment with latrunculin B at the concentration and exposure time used induced any obvious toxicity or cells death, as indicated by TUNEL staining and the total cell number (Fig. 3-1*B*,*C*).

10.1523/JNEUROSCI.0983-17.2017.f3-1 Figure 3-1 Confocal imaging of WGA stained cultures demonstrating a TNT formed between two astrocytes. The different layers (Z 01-Z 05) of the Z-stack (of the white rectangle) are shown to the right (A). A representative image of the TUNEL assay is shown in (B). Quantification of the number of TUNEL positive cells in relation to the total cell number reveled that there was less than 3 % TUNEL positive cells in all cell culture. Moreover, there was no significant difference in the percentage of TUNEL positive cells or the total cell number in cultures exposed to α-SYN oligomers or Latrunculin B, compared to untreated control cultures, at the used concentrations or exposure times (C). Scale bars (A) = 10 μm and (B) = 50μm. Data are presented as mean ± SD from three two experiments. The levels of significance were set to * P < 0.05, ** < 0.01 and *** < 0.001 (C). Download Figure 3-1, TIF file

### TNTs contribute to α-SYN transmission

**Figure 4.**
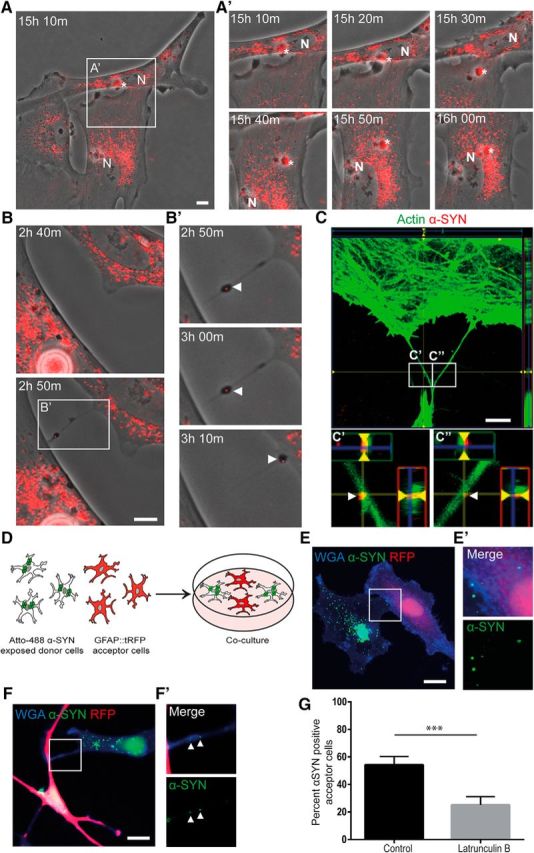
TNTs contribute to α-SYN transmission. Time-lapse experiments of α-SYN-Cy3 oligomer-exposed cultures showed that α-SYN was transported between the astrocytes in the culture via direct contact or via TNTs (see Fig. 4-1*A*). The different time points after α-SYN oligomer exposure are indicated. Direct transmission by membrane fusion of two astrocytes is shown in ***A***. Close-ups of the cell-to-cell contact region (white rectangle) demonstrate transmission of an α-SYN aggregate (white star) from the top cell to the bottom cell (15 h 20 min to 15 h 30 m after 24 h of α-SYN oligomer exposure) (***A′***). The α-SYN is then relocated toward the cell nuclei (N). Transfer of α-SYN-Cy3 via TNTs is shown in ***B***. The TNT formation was rather quick and took <10 min (2 h 40 min to 2 h 50 min) (***B***). Close-up imaging (white rectangle) over the after 20 min demonstrated clear TNT-mediated transfer of aggregated α-SYN (white arrowheads) (***B′***). Confocal microscopy confirmed the presence of α-SYN in actin-labeled TNTs (***C*** and Fig. 4-1*B*), close-up of the white rectangles are shown in ***C′*** and ***C″*** (white arrowheads indicate α-SYN). Cocultures of unlabeled astrocytes exposed to Atto 488-labeled α-SYN oligomers and untreated GFAP::tRFP astrocytes (***D*** and Fig. 4-2) demonstrated transfer of α-SYN to unaffected astrocytes via direct transfer (***E***) and TNTs (***F***). Close-ups of the cell-to-cell direct contact region and TNTs (white rectangles) are shown in ***E′*** and ***F′***, respectively (white arrowheads in ***F′*** indicate α-SYN). Latruculin B treatment reduced the percentage of α-SYN^+^ acceptor cells significantly (***G***). Scale bars: ***A***–***C***, 10 μm; ***E***, ***F***, 20 μm. Data are presented as mean ± SEM from three independent experiments and the levels of significance were set to *p < 0.05, **p < 0.01, and ***p < 0.001 (***G***).

Time-lapse experiments with Cy3-labeled α-SYN oligomer-exposed cultures demonstrated that α-SYN aggregates of different sizes (up to 5 μm in diameter) could transfer between the astrocytes in the culture (Fig. 4*A*,*B* and Fig. 4-1*A*, and Movies 1, 2, and 3). Direct transfer occurred between astrocytes that were in close contact (Fig. 4*A* and Movie 1, second transfer). Interestingly, we also noticed frequent transfer of α-SYN-Cy3 aggregates via thin cell protrusions, which resembled TNTs (Fig. 4-1*A*, and Movie 1, first transfer). Accordingly, time-lapse recordings also revealed transfer of α-SYN-Cy3 between the astrocytes via newly formed TNTs (Fig. 4*B* and Movies 2 and 3). Our time lapse-experiments indicated that very large inclusions of α-SYN were transported via direct cell contact, whereas smaller aggregates were easily transported via TNTs. Using confocal microscopy, we confirmed the presence of α-SYN-Cy3 within actin-labeled TNTs (Fig. 4*C* and Fig. 4-1*B*). Moreover, cocultures of unlabeled astrocytes exposed to α-SYN oligomers and untreated astrocytes expressing tRFP under the GFAP_ABC1D_ promotor (Holmqvist et al., 2015; Fig. 4*D* and Fig. 4-2) verified that α-SYN-containing astrocytes transferred α-SYN to unaffected astrocytes via direct cell contact (Fig. 4*E*) and via TNTs (Fig. 4*F*). The α-SYN TNT-mediated transmission was reduced significantly after pharmacological inhibition of actin polymerization using latrunculin B (Fig. 4*G*, *p* < 0.001). However, we cannot exclude that other transfer mechanisms are also affected by the latrunculin B treatment. Altogether, these data clearly demonstrate that astrocytic transfer of α-SYN occurs via TNTs *in vitro* and that pharmacological inhibition of actin polymerization prevents α-SYN transfer.

10.1523/JNEUROSCI.0983-17.2017.f4-1 Figure 4-1 Time lapse recordings demonstrated cell to cell spreading of α-SYN inclusions in the human astrocyte cultures. The astrocytes were exposed to Cy-3 labeled α-SYN oligomers for 24 h and then intensively washed prior to the experiment. Transfer occurred via thin TNT like cell protrusions. The first photo shows an overview and the following photos are close ups of the white rectangle. The different time points following α-SYN oligomer exposure are indicated at each photo and the α-SYN transfer is indicated with white stars. Higher magnifications of the TNT like cell protrusions (white arrows) are shown in the lowest panel (A). 3D confocal imaging confirmed the presence of Cy3-α-SYN in the TNTs (B). Scale bars (A) =10μm and (B) =2 μm. Download Figure 4-1, TIF file

10.1523/JNEUROSCI.0983-17.2017.f4-2 Figure 4-2 To study transfer between the human ES-derived astrocytes, co-cultures were performed with unlabeled astrocytes and astrocytes expressing tRFP under the GFAPABC1D promoter. The cell membrane marker WGA was used to identify all cells in the cultures. Scale bars = 50μm. Download Figure 4-2, TIF file

Movie 1. Time-lapse movie demonstrating that α-SYN-Cy3 (red, indicated with yellow arrow) are transferred from one astrocyte to another via thin, TNT-like cell protrusions (first transfer) and by close, membrane-to-membrane contact (second transfer). 10.1523/JNEUROSCI.0983-17.2017.video.1

Movie 2. Time-lapse movie demonstrating the formation of TNTs between two astrocytes (indicated with yellow arrow). 10.1523/JNEUROSCI.0983-17.2017.video.2

Movie 3. Close-up of Movie 2 demonstrating transfer of α-SYN-Cy3 (red, indicated with yellow arrow) from one astrocyte to another via the newly formed TNT. 10.1523/JNEUROSCI.0983-17.2017.video.3

### Storage of α-SYN results in ER swelling and impaired mitochondrial dynamics

**Figure 5.**
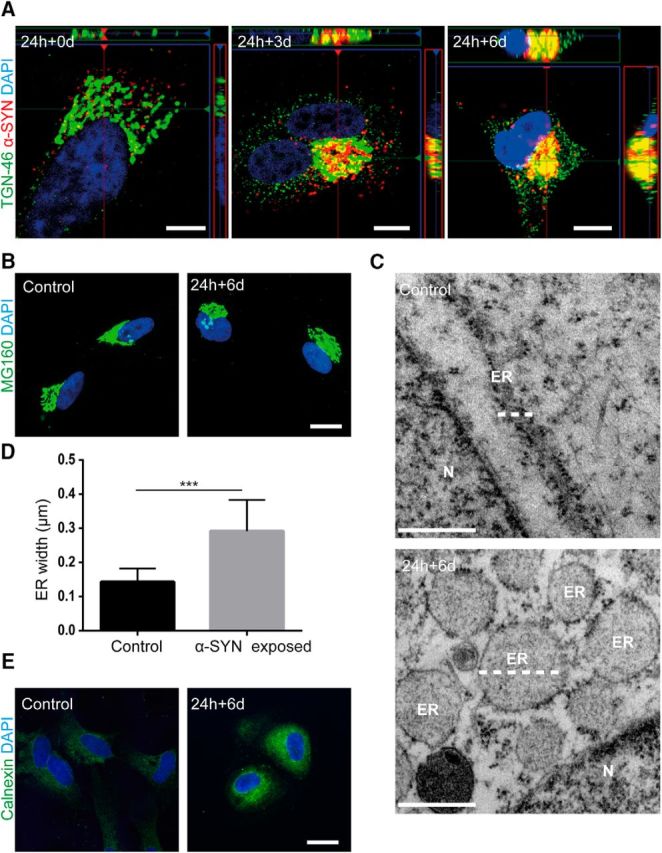
Intracellular storage of α-SYN in the TGN region causes ER swelling. Confocal microscopy imaging showed that α-SYN-Cy3 inclusions localized to the region of the TGN46^+^ TGN over time (***A*** and Fig. 5-1*A*). Immunostainings with specific anti-Golgi complex antibodies demonstrated that the accumulation of ingested α-SYN oligomers in the TGN region did not induce Golgi fragmentation (***B***). TEM analysis of astrocytes 6 d after α-SYN oligomer exposure (24 h + 6 d) indicated that the α-SYN storage induced ER swelling (***C***) (white dotted lines indicate ER). Quantification of the ER width confirmed the induced ER swelling in oligomer-exposed astrocytes (***D***). Representative images from immunostainings with the anti-calnexin antibody showed a higher expression of calnexin in the perinuclear region of α-SYN oligomer-exposed astrocytes compared with control (***E*** and Fig. 5-1*B*). Scale bars: ***A***, 10 μm; ***B***, 20 μm; ***C***, 500 nm; ***E***, 20 μm. Data are presented as mean ± SD from 14 α-SYN-exposed and 13 control astrocytes and the levels of significance were set to *p < 0.05, **p < 0.01, and ***p < 0.001 (***D***).

Immunocytochemistry followed by confocal microscopy demonstrated that the α-SYN-Cy3 inclusions were situated in the region of the trans-Golgi network (TGN) (Fig. 5*A* and Fig. 5-1*A*), but immunostaining with an anti-Golgi complex antibody indicated that the accumulation did not result in TGN fragmentation (Fig. 5*B*). However, from TEM analysis, it was obvious that the α-SYN accumulation resulted in severe defects in other cellular organelles. Six days after α-SYN oligomer exposure, the astrocytes displayed the typical appearance of ER swelling, with a pronounced increased volume of the ER lumen resembling a vesicular appearance (Fig. 5*C*). Quantification of the ER width in α-SYN oligomer-exposed cultures and untreated control cultures revealed that the α-SYN treatment increased the ER width significantly (*p* < 0.001; Fig. 5*D*). Furthermore, we performed immunostainings using specific antibodies to the ER-marker calnexin. The results from the immunostainings showed that there was a clearly higher expression of calnexin in the perinuclear region of α-SYN oligomers exposed astrocytes compared with control astrocytes (Fig. 5*E* and Fig. 5-1*B*).

10.1523/JNEUROSCI.0983-17.2017.f5-1 Figure 5-1 Separate channels of the Cy3-α-SYN and TGN-46 staining shown in Figure 5A (A). Separate channels of the Calnexin staining shown in Figure 5E (B). Scale bars: (A) = 10μm and (B) =20 μm. Download Figure 5-1, TIF file

**Figure 6.**
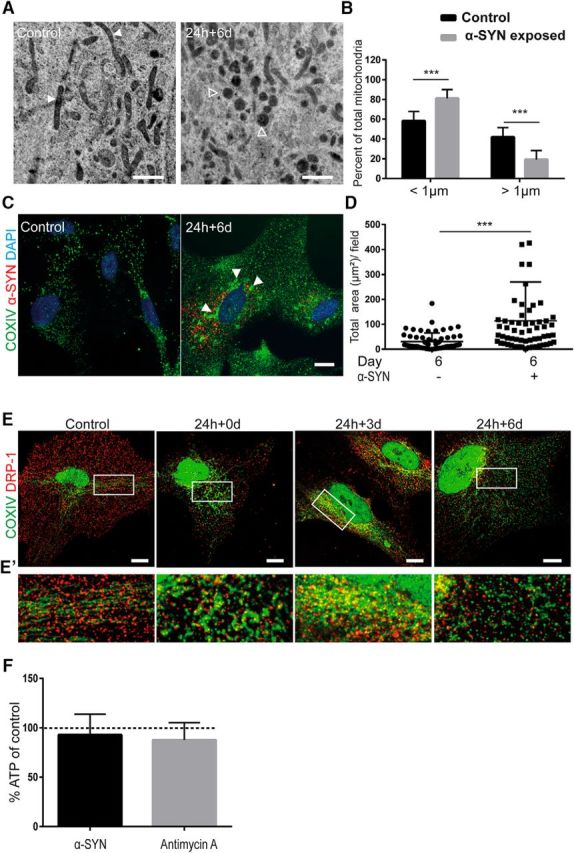
Accumulation of α-SYN oligomers results in mitochondrial defects and autophagy dysfunction. TEM analysis showed widespread mitochondrial fragmentation in α-SYN oligomer-exposed astrocytes compared with control cells (***A***) (white arrowheads indicate normal mitochondria and empty arrowheads indicate dense/fragmented mitochondria). Quantification of the percentage of normal mitochondria versus dense/fragmented mitochondria using ImageJ demonstrated a significant increase of dense mitochondria in oligomer-exposed cultures (***B***). Immunostaining with COXIV antibody demonstrated increased formation of mitochondria aggregates (***C*** and Fig. 6-1*A*, white arrowheads). The increased area of the mitochondria aggregates was confirmed by quantification using ImageJ analysis (***D***). Double staining with specific antibodies to COX IV and DRP-1 followed by confocal imaging displayed an organized pattern of mitochondria and the fission-regulating protein DRP-1 3 d after α-SYN oligomer exposure (24 h + 3 d), preceding an excessive amount of fragmented mitochondria at day 6 (24 h + 6 d) (***E*** and Fig. 6-1*B*). Close-ups of the white rectangles are shown in ***E′***. ATP measurement showed only a very moderate decrease (7%) in ATP levels after α-SYN oligomer treatment. In control cultures treated with the electron transport inhibitor antimycin A, there was also a very low decrease in ATP levels (13%) compared with untreated cultures (***F***). Scale bars: ***A***, 1 μm; ***C***, ***E***, 20 μm. Data are presented as mean ± SD from 14 α-SYN oligomer-exposed and 12 control astrocytes (***B***) and as mean ± SD from three independent experiments (***D***). The levels of significance were set to *p < 0.05, **p < 0.01, and ***p < 0.001 (***B***, ***D***).

Moreover, α-SYN oligomer-exposed cultures displayed altered mitochondrial morphology, with an elevated number of small and dense mitochondria (Fig. 6*A*). To be able to quantify the effect on mitochondrial fusion/fission, we divided the mitochondria into two groups depending on their length and their dense/fragmented morphologies (the normal mitochondria were >1 μm, whereas small/dense mitochondria were <1 μm). Quantification of the number of mitochondria in the two groups showed that α-SYN oligomer exposure increased the percentage of small and dense mitochondria significantly (*p* < 0.001) and decreased the percentage of normal mitochondria (*p* < 0.001; Fig. 6*B*). Immunostaining with the mitochondrial marker COXIV demonstrated pathological clusters of mitochondria in the astrocytes, further verifying the disrupted mitochondrial dynamics (Fig. 6*C* and Fig. 6-1*A*). Quantification of the total area of the COXIV^+^ regions in astrocytes at day 6 demonstrated that the presence of mitochondrial clusters increased significantly after α-SYN oligomer exposure (Fig. 6*D*). Double staining using specific antibodies to COXIV and DRP-1 followed by confocal imaging revealed an organized pattern of mitochondria and the fission-regulating protein 0 and 3 d after α-SYN oligomer exposure, preceding the excessive amount of fragmented mitochondria at day 6 (Fig. 6*E*,*E′* and Fig. 6-1*B*). These results indicate that the mitochondrial clusters observed in Figure 6, *C* and *D*, may be due, at least in part, to increased mitochondrial fragmentation. Interestingly, ATP measurement showed only a very slight decrease (7%) in ATP levels after α-SYN oligomer treatment. In control cultures treated with the electron transport inhibitor antimycin A, there was also a very modest decrease in ATP levels (13%) compared with untreated cultures (Fig. 6*F*). These results, together with the low apoptotic frequency after α-SYN oligomer exposure observed with the TUNEL assays (Fig. 3-1*B*), indicate that the astrocytes somehow are able to compensate for the substantial mitochondrial damage.

10.1523/JNEUROSCI.0983-17.2017.f6-1 Figure 6-1 Separate channels of the Cy3-α-SYN and COXIV staining shown in Figure 5C (A). Separate channels of the DRP-1 and COXIV staining shown in Figure 6 E. Close ups from the white rectangles are shown below. Scale bars: (A) = 20μm and (B) = 10μm. Download Figure 6-1, TIF file

### Accumulation of α-SYN affects the autophagosomal machinery

**Figure 7.**
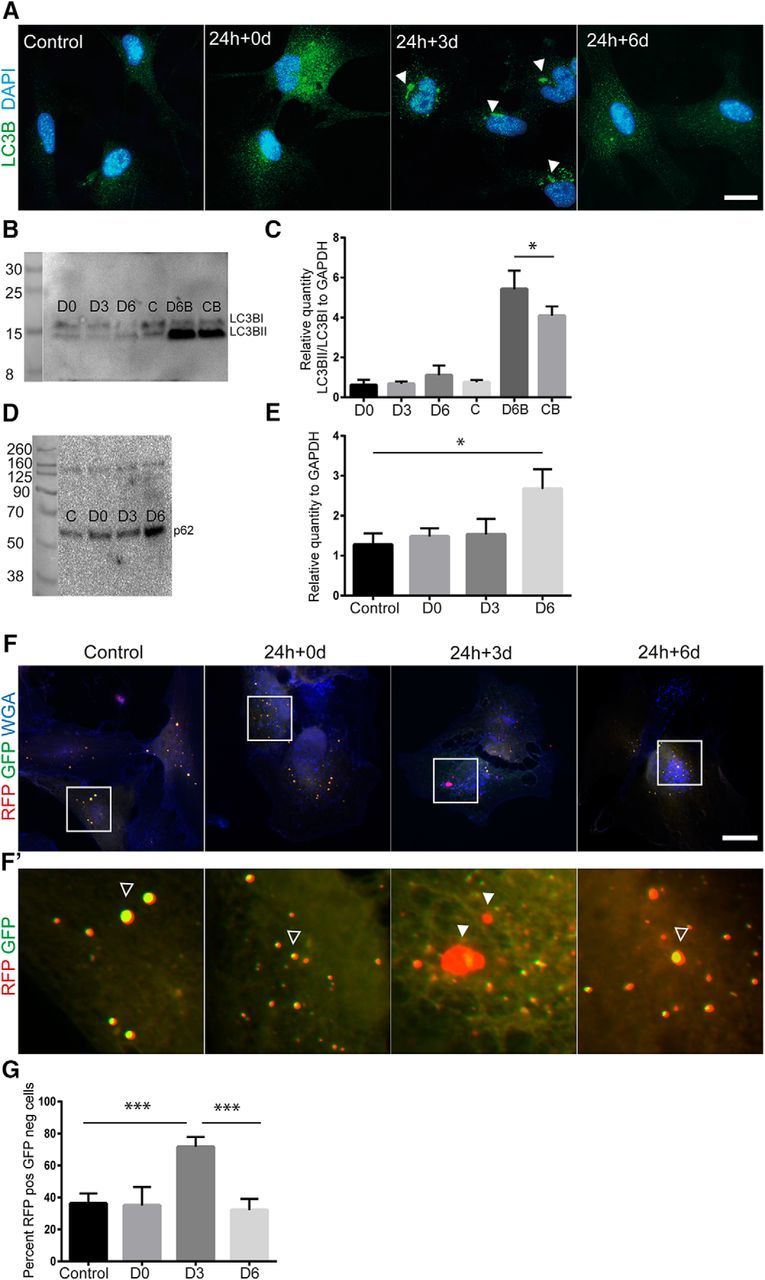
α-SYN oligomer exposure affects the autophagosomal pathway. Immunostainings with specific antibodies to the autophagy marker LC3B showed an increased presence of LC3B puncta after α-SYN oligomer exposure (24 h + 0 and 24 h + 3 d) compared with untreated control cultures (***A***). Western blot analysis of LC3BI and II showed a significantly increased LC3BII/I ratio (normalized to GAPDH) at 24 h + 6 d when the cells were treated with the inhibitor bafilomycin A, suggesting a partial block of the autophagosomal machinery by the α-SYN oligomers (***B***, ***C***). Western blot analysis of p62 showed a significant increased protein expression at day 6 after oligomer exposure (24 h + 6 d) compared with untreated controls, (*n* = 3) (***D***, ***E***). Next, astrocytes were transfected with plasmids containing the LC3B gene coupled to RFP and pH-sensitive GFP. This autophagy tandem sensor RFP–GFP technique makes it possible to follow the turnover of autophagosomes to autolysosomes because the GFP protein is degraded when it comes into contact with low pH. Representative images from the staining demonstrate that significantly more cells with RFP^+^ but GFP^−^ autophagosomes were present at 24 h+3 d compared with untreated control cultures (***F***–***H*** and Fig. 7-1) Filled arrowheads indicate RFP^+^ GPP^−^ vesicles and empty arrowheads indicate RFP^+^ GPP^+^ vesicles. Scale bars: ***A***, ***E***, 20 μm. Data are presented as mean ± SD.

Normally, defected mitochondria and protein aggregates are degraded and removed from the cell by autophagy. When autophagy is induced, the autophagosome is formed by conjugation of cytosolic LC3B I (17 kDa) to phosphotidylethanolamine, forming LC3BII (15 kDa), which is recruited to the autophagosomal membrane by p62 (Katsuragi et al., 2015). The autophagosome then fuses with the lysosome to form the autolysosome in which the low pH will facilitate degradation of the material. During the fusion with the lysosomes, the low pH also degrades the LC3BII and p62 proteins. To investigate whether the increased number of fragmented mitochondria after α-SYN exposure was due to ineffective mitophagy, we next examined various steps of the autophagy pathway, including formation of LC3B puncta, LC3BII/I ratio, p62 expression, and autophagosomal turnover. Because cytoplasmic LC3 is processed and recruited to the autophagosomal membranes, it is possible to identify cells undergoing autophagy by visualizing fluorescently labeled LC3 puncta. We could indeed identify the formation of LC3^+^ puncta in αSYN-treated cultures (Fig. 7*A*). Interestingly, by day 6, the autophagosomes were reduced to normal levels, indicating that the autophagosomal pathway was initially induced but then halted, although the fragmented mitochondria were still present (Fig. 7*A*). To investigate whether α-SYN oligomer exposure affects autolysosomal formation and degradation, cells were exposed to bafilomycin A, which inhibits the formation of the autolysosome and neutralizes the lysosomal pH, leading to less degradation. This treatment will then cause accumulation of the LC3II and p62 proteins because the proteins will not be degraded by the autolysosomes. Interestingly, the α-SYN and bafilomycin-treated cells showed a significantly higher LC3II/I ratio compared with cultures treated with bafilomycin only. These data demonstrate that the αSYN oligomers interfere with the autophagosome/lysosome fusion and partially block the autophagic flux (Klionsky et al., 2016; Fig. 7*B*,*C*). Furthermore, Western blot analysis showed a significant increase in the expression of p62 in astrocyte cultures at day 6 after αSYN oligomer exposure, indicating that the αSYN oligomers interfere with the autophagosome/lysosome fusion, which is in consistent with the LC3II results (Fig. 7*C*,*D*). Using an autophagy tandem sensor RFP-GFP kit, we transfected the astrocytes with plasmids containing the LC3B gene coupled to RFP- and pH-sensitive GFP. This technique makes it possible to follow the turnover of autophagosomes to autolysosomes because the GFP protein is degraded when it comes into contact with low pH. Interestingly, we found a significantly higher number of astrocytes with RFP^+^ but GFP^−^ vesicles at day 3 after αSYN oligomer exposure, demonstrating that there is an upregulation of autolysosome formation at this time point. However, at day 6 after αSYN oligomer exposure, the number of astrocytes with RFP^+^ GFP^−^ vesicles were similar to control cultures (Fig. 7*F–H* and Fig. 7-1). Together, these results suggest that the α-SYN oligomer exposure induces activation of the autophagosomal machinery, but that the autophagy is discontinued even though the degradation is not completed.

10.1523/JNEUROSCI.0983-17.2017.f7-1 Figure 7-1 Separate channels from the LC3B-RFP-GFP staining shown in Figure 7 F (A). As a control chlouroscin was added to the cells, which neutralizes lysosomal pH and inhibits the degradation of the pH sensitive GFP protein (B). Scale bars: (A and B) = 20 μm. Download Figure 7-1, TIF file

### Healthy astrocytes transmit mitochondria to stressed α-SYN-containing astrocytes

**Figure 8.**
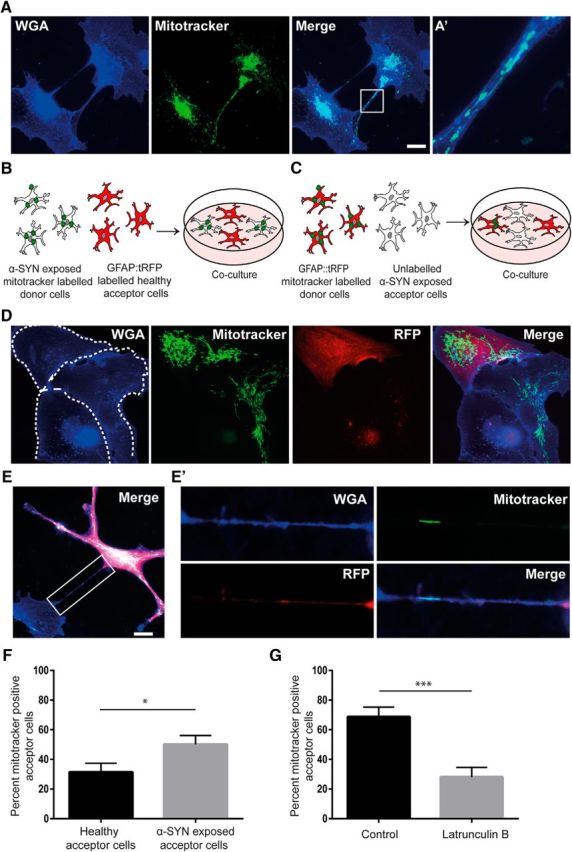
Healthy astrocytes deliver mitochondria to α-SYN oligomer-exposed astrocytes. Using Mitotracker transfection, we could demonstrate that mitochondria were present within TNTs connecting the astrocytes (***A***). A close-up of the white rectangle is shown in ***A′***. Coculture experiments were performed using two different set-ups. Either, GFAP::tRFP astrocytes (red acceptor cells) were cocultured with Mitotracker-transfected, α-SYN oligomer-exposed astrocytes (donor cells) (***B***) or α-SYN oligomer-exposed astrocytes (acceptor cells) were cocultured with GFAP::tRFP, Mitotracker-transfected astrocytes (red donor cells) (***C***). Using immunocytochemistry, we identified transfer of mitochondria between the GFAP::tRFP astrocytes and the unlabeled astrocytes via direct contact (dashed areas show the outline of separate cells) (***D***) and TNTs (***E***), a close-up of the white rectangle is shown in ***E′***. Quantification of the number of Mitotracker^+^ acceptor cells in the two coculture setups indicated that the healthy astrocytes transferred significantly more mitochondria to α-SYN oligomer-exposed cells than the other way around (***F***). Addition of latrunculin B to the cocultures significantly decreased the percentage of Mitotracker^+^ acceptor cells (***G***). In addition, basal level of mitochondrial transfer was investigated in untreated control cultures. Compared with α-SYN oligomer-exposed cultures, the number of Mitotracker^+^ acceptor cells was significantly lower in the control cultures (see Fig. 8-1). Scale bars: ***A***, 20 μm; ***D***, ***E***, 20 μm. Data are presented as mean ± SEM from three independent experiments and the levels of significance were set to *p < 0.05, **p < 0.01, and ***p < 0.001 (***F***, ***G***).

It was shown recently that mitochondria could transfer via TNTs to rescue “injured” cells (Wang and Gerdes, 2015). Due to the supporting nature of astrocytes in the brain, we wanted to test whether this phenomenon also takes place in α-SYN oligomer-exposed human astrocytes. By labeling the mitochondria with Mitotracker, we could identify transfer of mitochondria between the astrocytes in α-SYN-treated cultures (Fig. 8*A*). Next, we sought to investigate whether healthy astrocytes could transfer mitochondria to α-SYN oligomer-exposed astrocytes. To this end, we performed two different coculture experiments. In one setup, RFP-expressing astrocytes (acceptor cells) were cocultured with Mitotracker-labeled, α-SYN oligomer-exposed astrocytes (donor cells) (Fig. 8*B*). In the other setup, α-SYN oligomer-exposed astrocytes (acceptor cells) were cocultured with RFP-expressing, Mitotracker-labeled astrocytes (donor cells) (Fig. 8*C*). Using this approach, we could clearly identify transfer of Mitotracker-labeled mitochondria between the untreated and the α-SYN oligomer-exposed astrocytes via both direct transfer (Fig. 8*D*) and TNTs (Fig. 8*E*). Quantification of the number of acceptor cells in both experimental setups indicated that the healthy astrocytes transferred significantly more mitochondria to α-SYN oligomer-exposed cells than the other way around (Fig. 8*F*, *p* < 0.05). The addition of latrunculin B to the cocultures resulted in a >2-fold decrease in the number of acceptor astrocytes (Fig. 8*G*, *p* < 0.001). In cocultures in which neither the acceptor cells nor the donor cells were treated with α-SYN oligomers, the level of mitochondrial transfer was significantly lower than in α-SYN oligomer-exposed cultures (*p* < 0.001) (Fig. 8-1).

10.1523/JNEUROSCI.0983-17.2017.f8-1 Figure 8-1 Basal level of mitochondrial transfer was investigated in untreated control cultures. In comparison to α-SYN oligomer exposed cultures the number of Mitotracker positive acceptor cells was significantly lower in the control cultures. Data are presented as mean ± SEM from three independent experiments. The levels of significance were set to * P < 0.05, ** < 0.01 and *** < 0.001. Download Figure 8-1, TIF file

## Discussion

Compelling experimental data suggest that the transfer of toxic α-SYN species from affected cells to healthy cells results in the anatomical spread of pathology seen during PD progression (Recasens and Dehay, 2014). However, the cell types responsible for the α-SYN propagation and the exact transmission mechanisms remain to be elucidated. In a recent study, Abounit et al. (2016) demonstrated that preformed α-SYN fibrils could transfer between cultured mouse neurons via TNTs. However, no study has yet demonstrated whether such transfer occurs directly between bona fide human glia or human neurons and human glia. Here, we report for the first time that aggregated α-SYN can be transferred between human astrocytes via direct contact and newly formed TNTs and that this transfer can be abrogated using the pharmacological agent latrunculin B. Previous evidence for TNTs in intercellular transfer of misfolded proteins comes primarily from studies of prion diseases (Gousset et al., 2009). For example, the prion protein (PrP) can spread from infected to uninfected neurons or from dendritic cells to uninfected neurons (Gousset et al., 2009). Interestingly, astrocytes that display early PrP accumulation in affected brains have been shown to transfer PrP effectively to uninfected neurons via cell-to-cell contact (Victoria et al., 2016). In addition to PrP, TNT-mediated transfer of Aβ has been demonstrated to occur between primary rodent astrocytes (Wang et al., 2011).

Increasing evidence emphasizes that insufficient lysosomal degradation is involved in the pathogenesis of different neurodegenerative diseases, including PD (Nixon, 2007; Nixon et al., 2008; Appelqvist et al., 2013). Accordingly, it is known that patients with lysosomal storage disorders (LSDs) often develop neurodegenerative diseases (Nixon, 2007; Appelqvist et al., 2013). For LSDs such as multiple sulfatase deficiency and Gaucher disease, it has been shown that normal autophagic, endocytic, and lysosomal vesicle trafficking can be essential for preventing neurodegeneration (Mazzulli et al., 2011; Di Malta et al., 2012). Here, we show that α-SYN oligomers, after engulfment by human astrocytes, colocalized with the endosomal–lysosomal marker LAMP-1. However, although the intracellular deposits of ingested α-SYN oligomers persisted, the colocalization with LAMP-1 declined drastically over time. In contrast to the oligomers, α-SYN monomers became fully degraded by the human astrocytes. These observations raised the question of whether the oligomers affect the lysosomal machinery directly or if the α-SYN accumulation is due to an overall slow degradation of heavily compacted material by the astrocytes. Our observations, together with previous studies, indicate that it is probably a combination of both.

We have demonstrated previously that astrocytes engulf large amounts of aggregated Aβ_42_ that are stored in the cells for a very long time (Söllvander et al., 2016). Similarly, we have shown that astrocytes effectively engulf dead cells both *in vitro* and *in vivo* and they are degraded very slowly (Lööv et al., 2012). A general slow digestion in astrocytes may be explained by their antigen presentation properties. Several studies have suggested that astrocytes are involved in T-cell activation because they express major histocompatibility complex class II (Cornet et al., 2000; Lööv et al., 2015). Accordingly, T-cell infiltration has been observed in the PD brain at advanced disease stages. Conversely, aggregated α-SYN has been shown to affect lysosomal function by inhibiting the activity of lysosomal enzymes (Mazzulli et al., 2011). Interestingly, lysosomal dysfunction in SH-SY5Y cells increases α-SYN release via exosomes (Alvarez-Erviti et al., 2011). Moreover, a recent study demonstrated that lysosomes containing preformed α-SYN fibrils can transfer between neuronal cells (Abounit et al., 2016). In this study, we did not observe any intercellular transfer of α-SYN situated within lysosomes, which could be explained by the fact that astrocytes store the α-SYN in LAMP-1^−^ compartments. The incomplete degradation of ingested α-SYN oligomers in human astrocytes leads to intracellular accumulation in the TGN region. Retrograde transport of proteins from endosomes to the TGN is a well known phenomenon (Bonifacino and Rojas, 2006). Localization of α-SYN deposits in TGN has been demonstrated previously to cause Golgi fragmentation, impaired vesicular transport, blockage of ER–Golgi transport (Cooper et al., 2006), and lysosomal dysfunction in dopaminergic neurons (Mazzulli et al., 2016). The α-SYN oligomer-exposed human astrocytes showed no signs of Golgi fragmentation or increased apoptosis, but displayed severe ER swelling and autophagy disturbances, as well as mitochondria impairment, indicating that the astrocytes were substantially stressed.

There have been several hypotheses about how α-SYN aggregates induce mitochondrial dysfunction in neurons. Among these, disturbance of the mitochondria fission–fusion hemostasis, autophagososme synthesis, and mitophagy function in particular has been highlighted (Gui et al., 2012; Nakamura, 2013; Guardia-Laguarta et al., 2014; Ryan et al., 2015). A functional autophagosomal pathway is highly relevant to prevent neurodegeneration because it is a key route for the degradation of a range of intracytoplasmic aggregate-prone proteins and is also a disposal route for dysfunctional mitochondria. Our data demonstrate a widespread mitochondrial fragmentation in the α-SYN-treated astrocytes and suggest that the accumulation of α-SYN aggregates in the TGN region may disrupt the autophagosomal and mitophagy machinery because the autophagosomal membranes are provided by the ER and Golgi. Moreover, our examinations of various steps in their autophagosomal pathway indicate that autophagy is induced but then halted, although the fragmented mitochondria are still present. These findings indicate that the accumulation of α-SYN in the astrocytes disrupts their lysosomal machinery and prevents clearance of the damaged mitochondria. Consistent with our findings, it has been shown previously that overexpression of α-SYN can inhibit autophagosome formation (Winslow et al., 2010).

As a consequence of the ineffective degradation of α-SYN oligomers and mitochondrial disturbances, the stressed astrocytes deploy a “defense mechanism” that consists of sending out TNTs to other astrocytes, enabling direct intercellular transfer of the toxic protein to healthy astrocytes. In return, healthy astrocytes send mitochondria to rescue the stressed, α-SYN-accumulating astrocytes, suggesting that astrocytes naturally help each other by compensating for protein aggregation and cellular stress with new energy-producing mitochondria. This finding is consistent with earlier observations that TNT-mediated transfer of mitochondria from healthy cells can rescue UV-treated cells from apoptosis (Spees et al., 2006; Wang and Gerdes, 2015; Han et al., 2016; Jiang et al., 2016). Moreover, it was shown recently that mesenchymal stem cells can transfer their mitochondria to macrophages via TNTs and thereby enhance macrophage phagocytosis of bacteria (Jackson et al., 2016).

**Figure 9.**
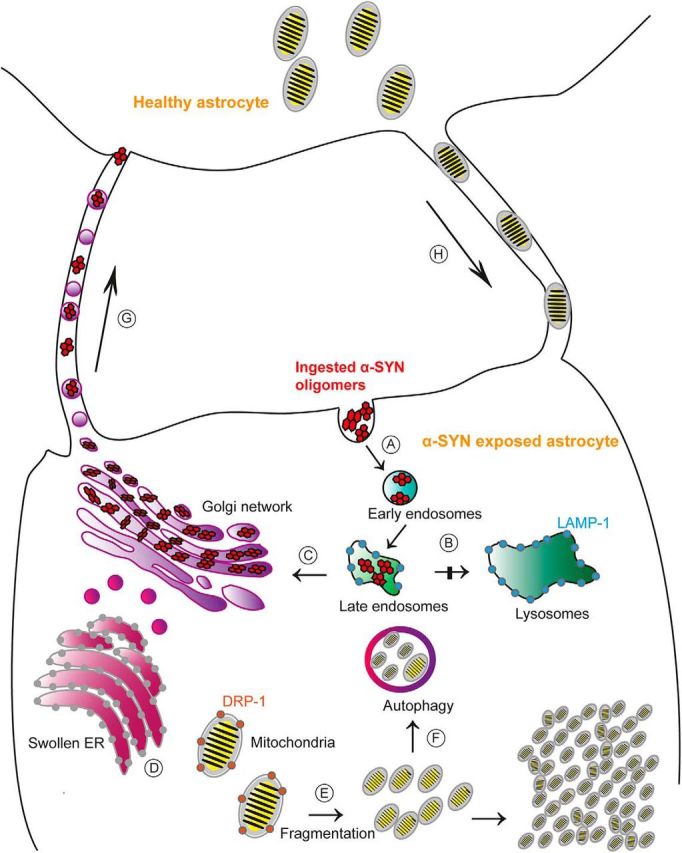
Proposed model for the cellular effects of α-SYN accumulation in astrocytes. Ingested α-SYN oligomers are transported to early/late endsosomes (***A***), but the lysosomal degradation is not completed (***B***). Instead, the aggregated α-SYN is stored intracellularly in the TGN region (***C***). The α-SYN accumulation is clearly stressful for the cell and results in swollen ER (***D***) and mitochondria fragmentation (***E***). Impaired mitochondria are initially degraded by the autophagosomes (***F***). However, the mitophagy is insufficient and pathological mitochondria remain in the astrocyte. Moreover, α-SYN oligomer-exposed astrocytes rapidly deploy a “defense mechanism” that consists of sending out TNTs to other astrocytes, enabling direct intercellular transmission of the toxic protein to healthy astrocytes (***G***). In return, healthy astrocytes send mitochondria to “rescue” stressed astrocytes (***H***).

Neurodegenerative diseases, including PD, are defined by loss of brain homeostasis (Giaume et al., 2007; Coulter and Eid, 2012; Verkhratsky et al., 2012; Verkhratsky et al., 2013; Verkhratsky et al., 2015), which could be explained, at least in part, by the fact that severely stressed astrocytes are unable to fulfill their normal tasks (Eroglu and Barres, 2010; Sofroniew and Vinters, 2010; Verkhratsky et al., 2015). In conclusion, our data show that high loads of α-SYN in human astrocytes induced ER swelling, lysosomal–autophagosomal dysfunction, and consequently TNT-mediated transfer of aggregated α-SYN and mitochondria (Fig. 9). Using a pharmacological approach to inhibit TNT formation, we abolished the transfer of both α-SYN and mitochondria, identifying astrocytic TNTs as a possible drug target.
