# Supplementary figures and images for: Human Astrocytes Transfer Aggregated Alpha-Synuclein via Tunneling Nanotubes
Source: J Neurosci. 2017 Dec 6;37(49):11835–53. doi: 10.1523/JNEUROSCI.0983-17.2017 (PMC5719970; doi:10.1523/JNEUROSCI.0983-17.2017)

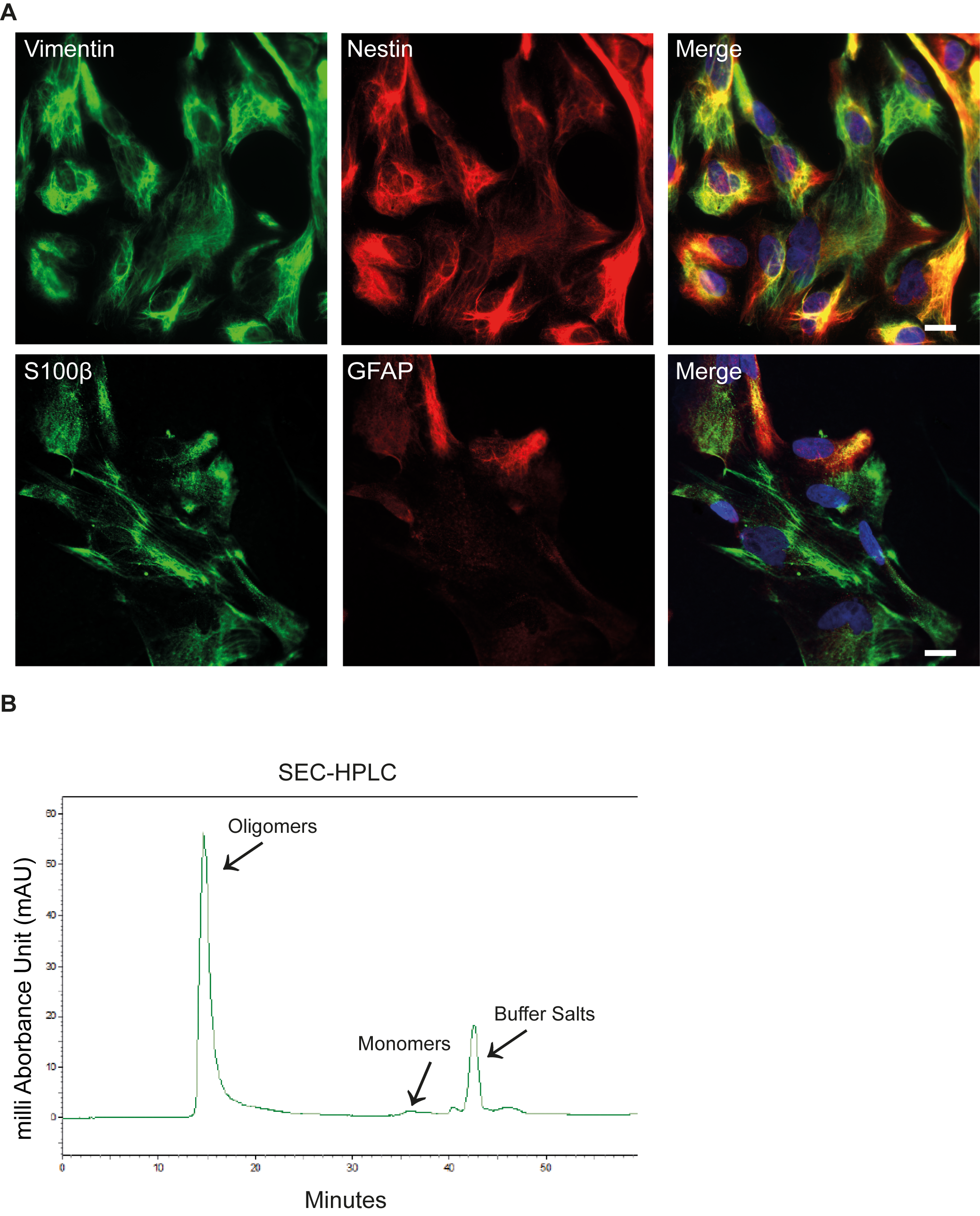

Supplement: Figure 1-1 [file zns999170335so1.tif]

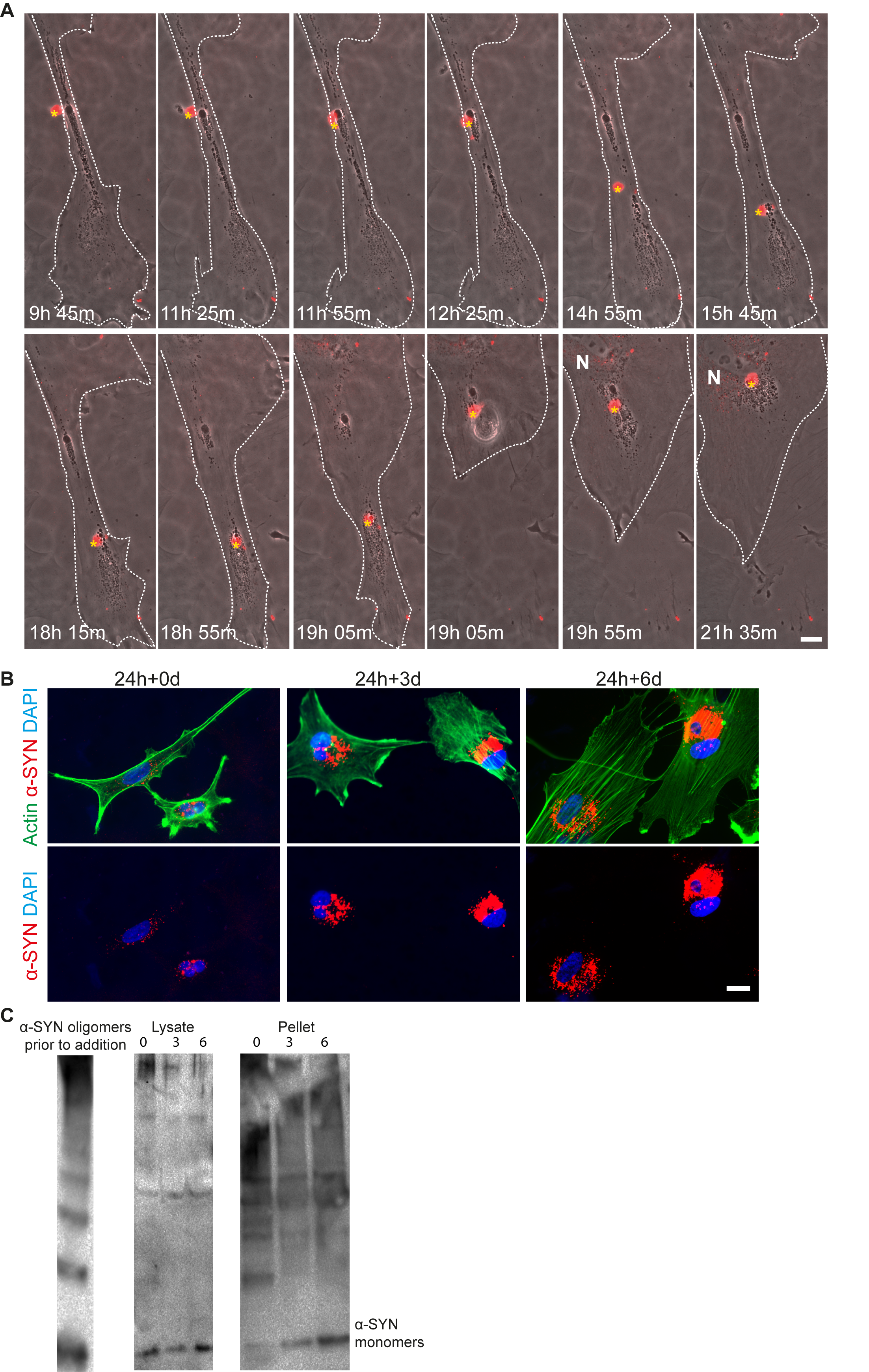

Supplement: Figure 1-2 [file zns999170335so2.tif]

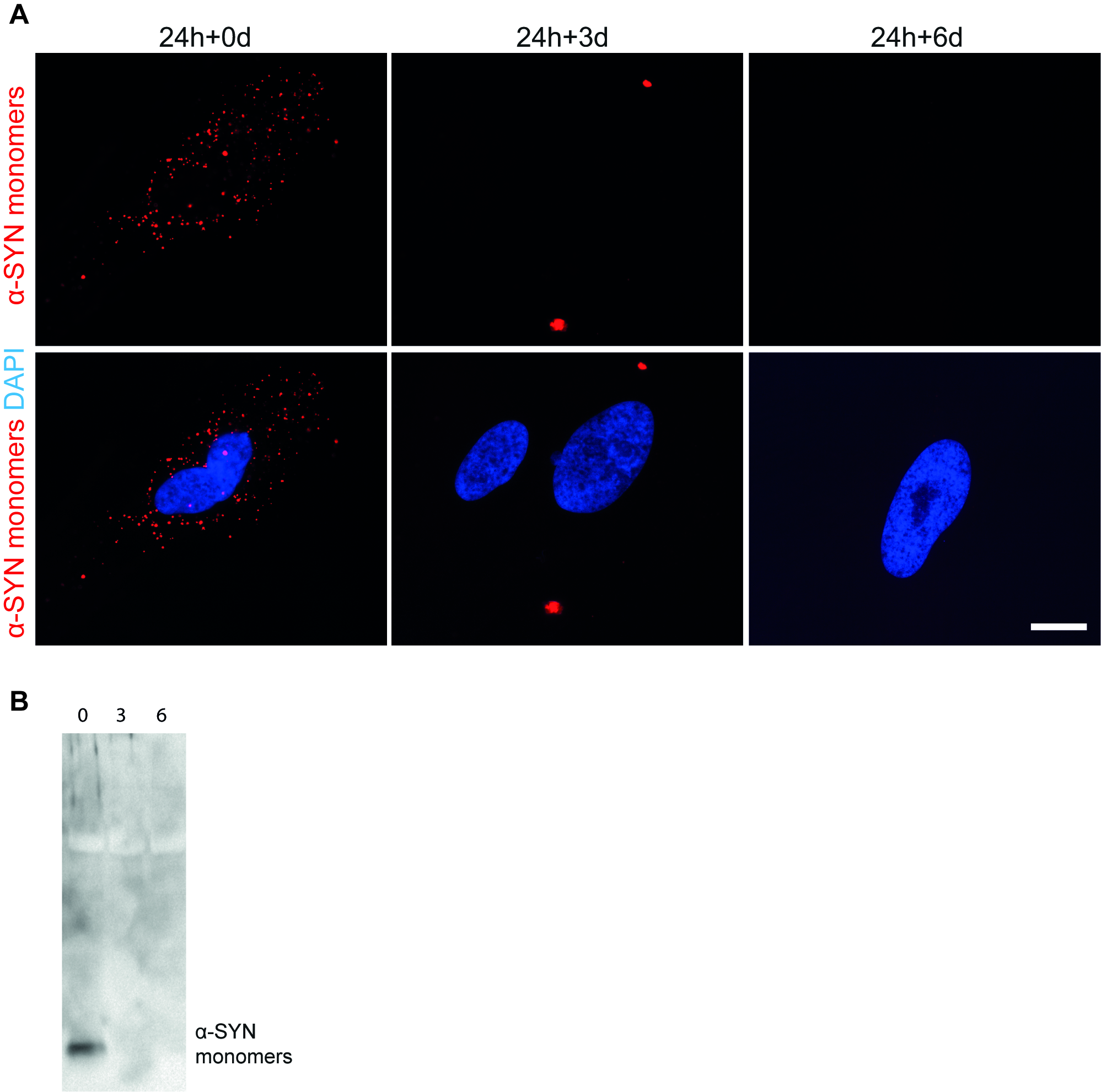

Supplement: Figure 1-3 [file zns999170335so3.tif]

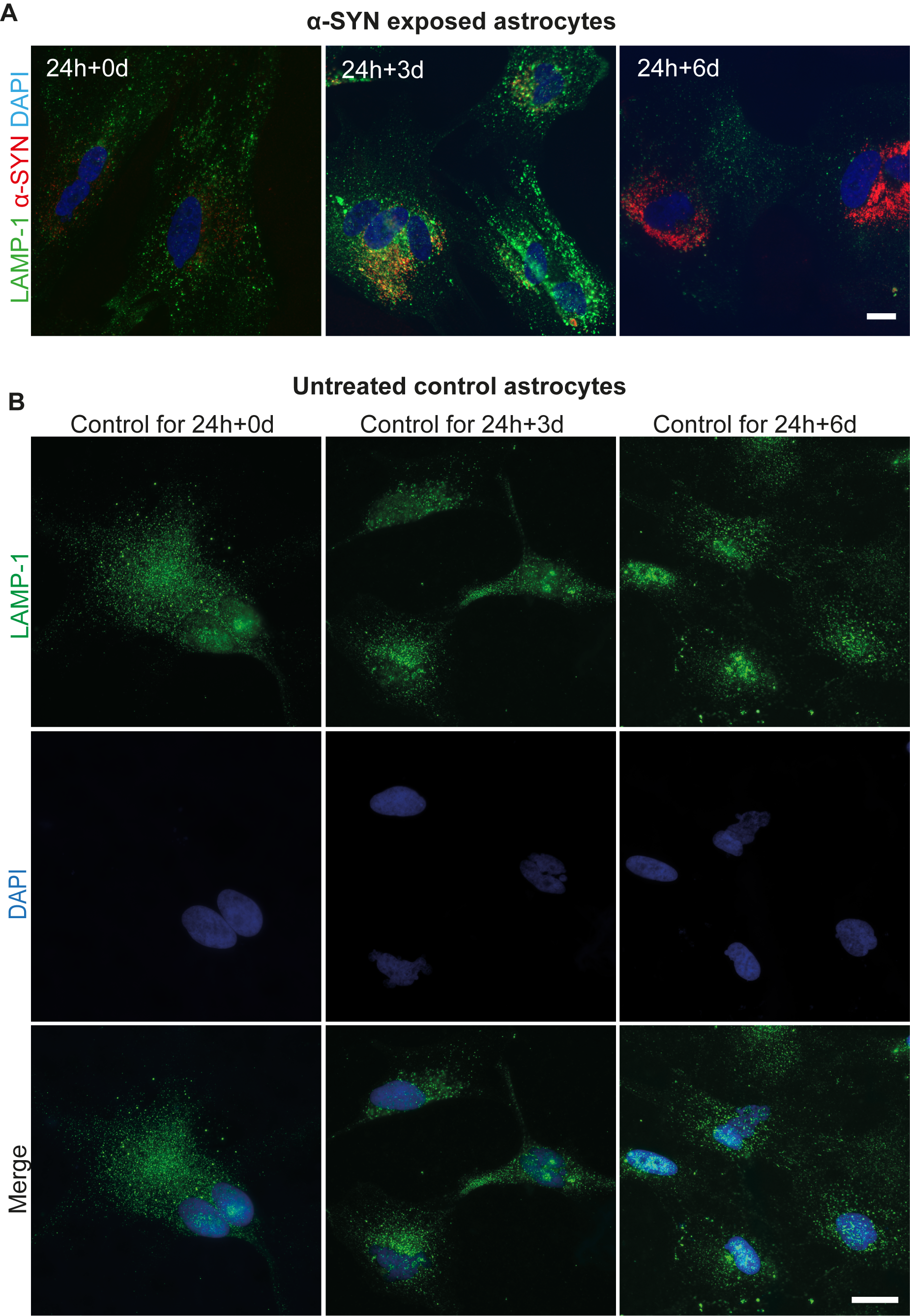

Supplement: Figure 2-1 [file zns999170335so4.tif]

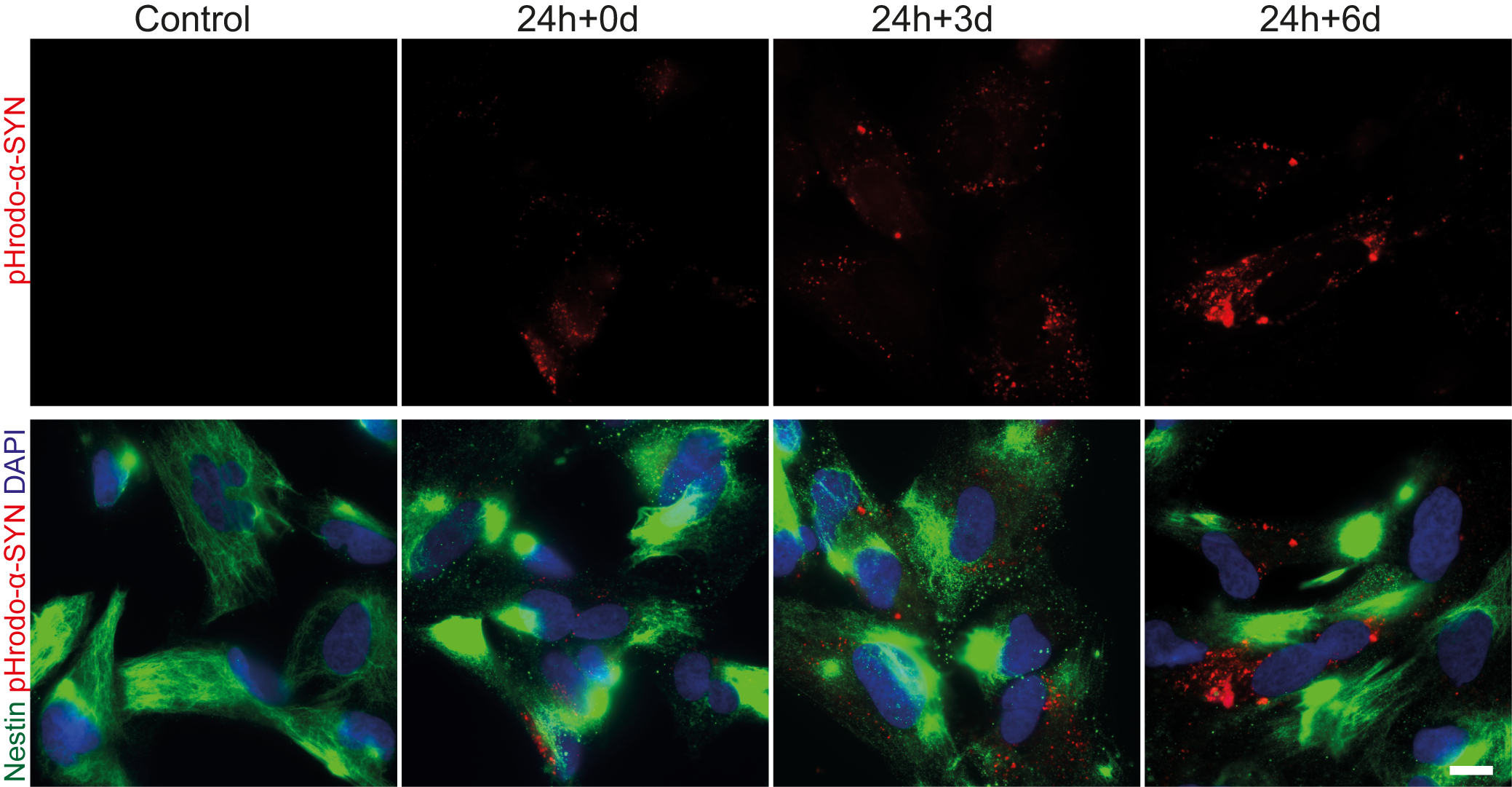

Supplement: Figure 2-2 [file zns999170335so5.tif]

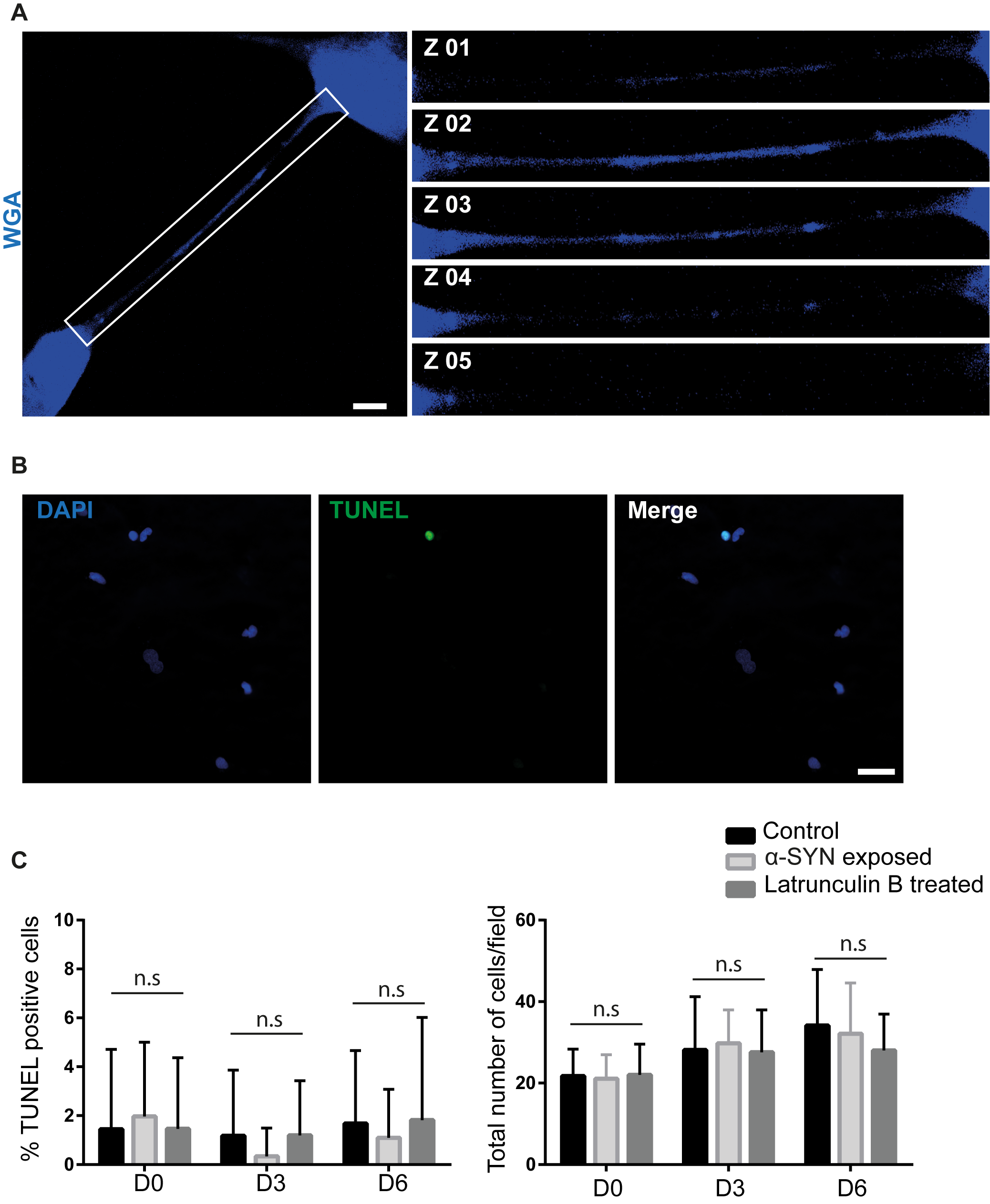

Supplement: Figure 3-1 [file zns999170335so6.tif]

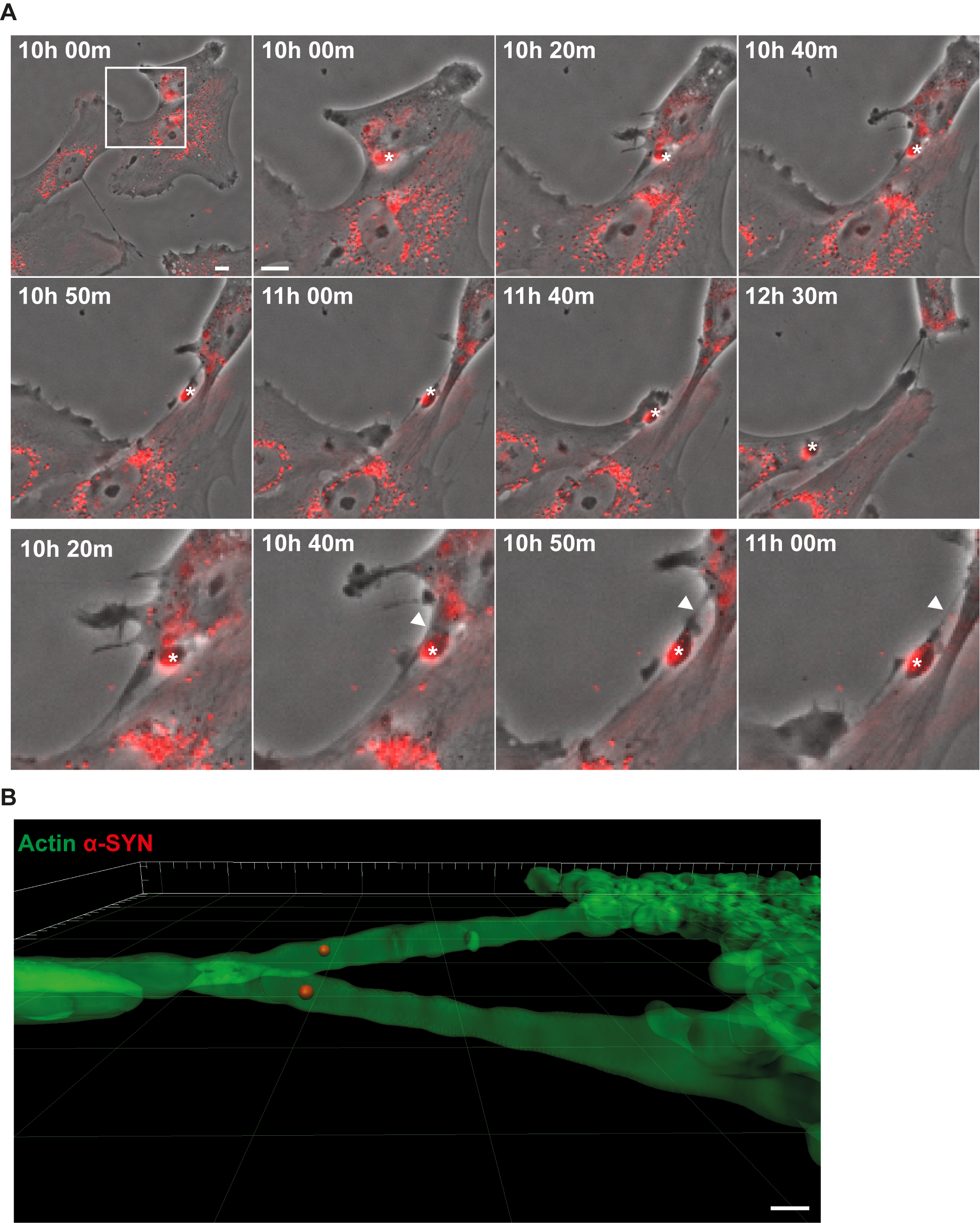

Supplement: Figure 4-1 [file zns999170335so7.tif]

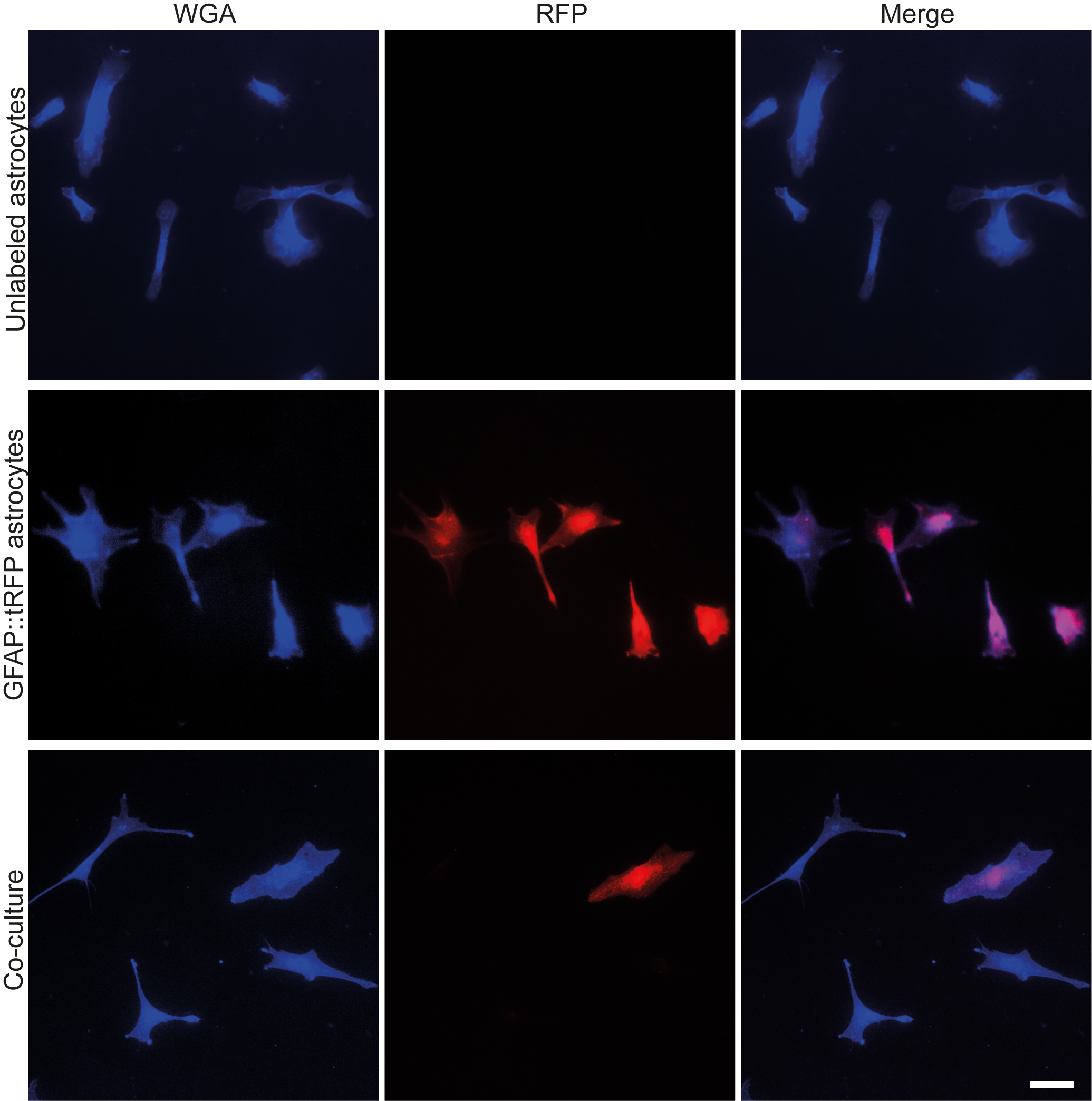

Supplement: Figure 4-2 [file zns999170335so8.tif]

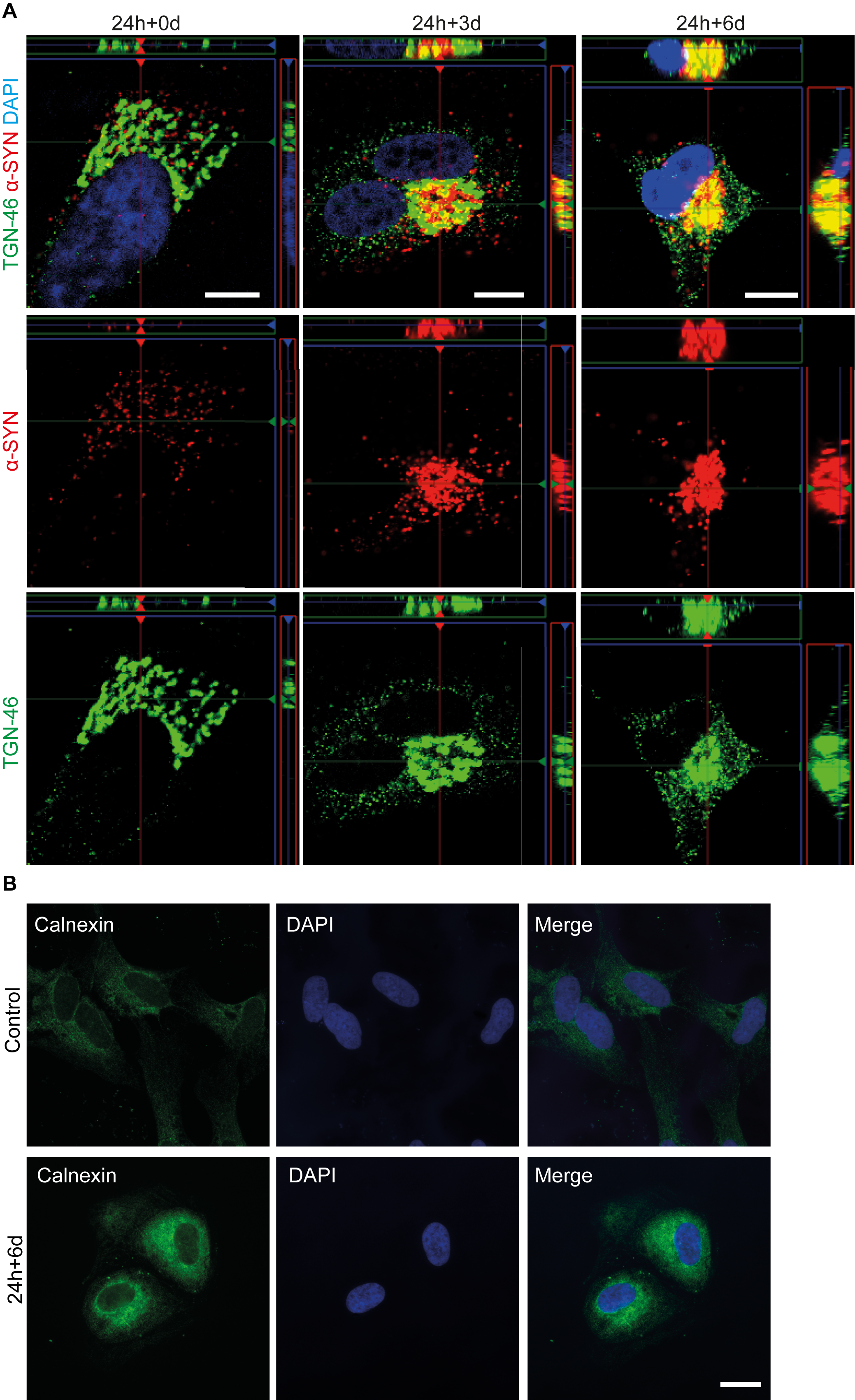

Supplement: Figure 5-1 [file zns999170335so9.tif]

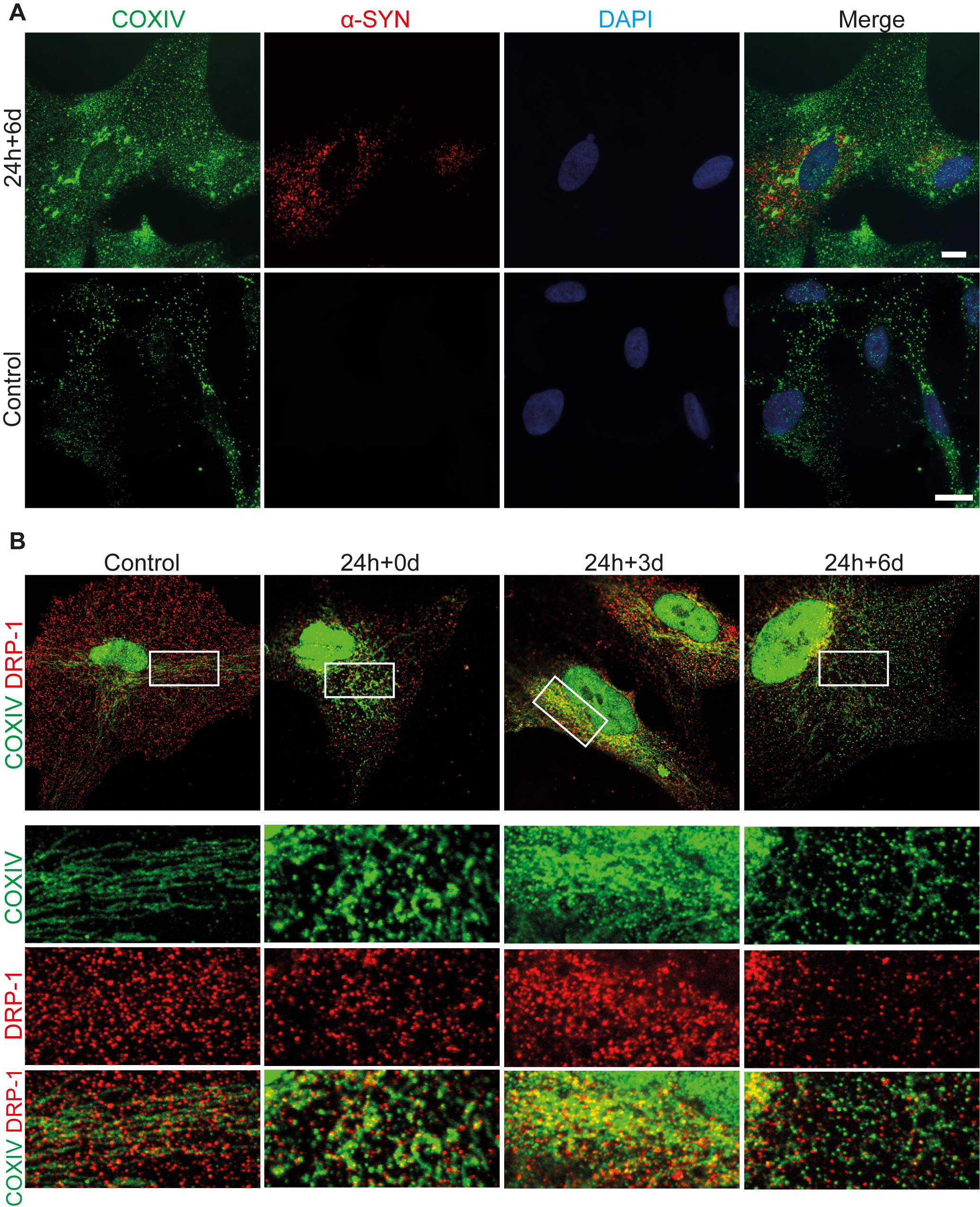

Supplement: Figure 6-1 [file zns999170335so10.tif]

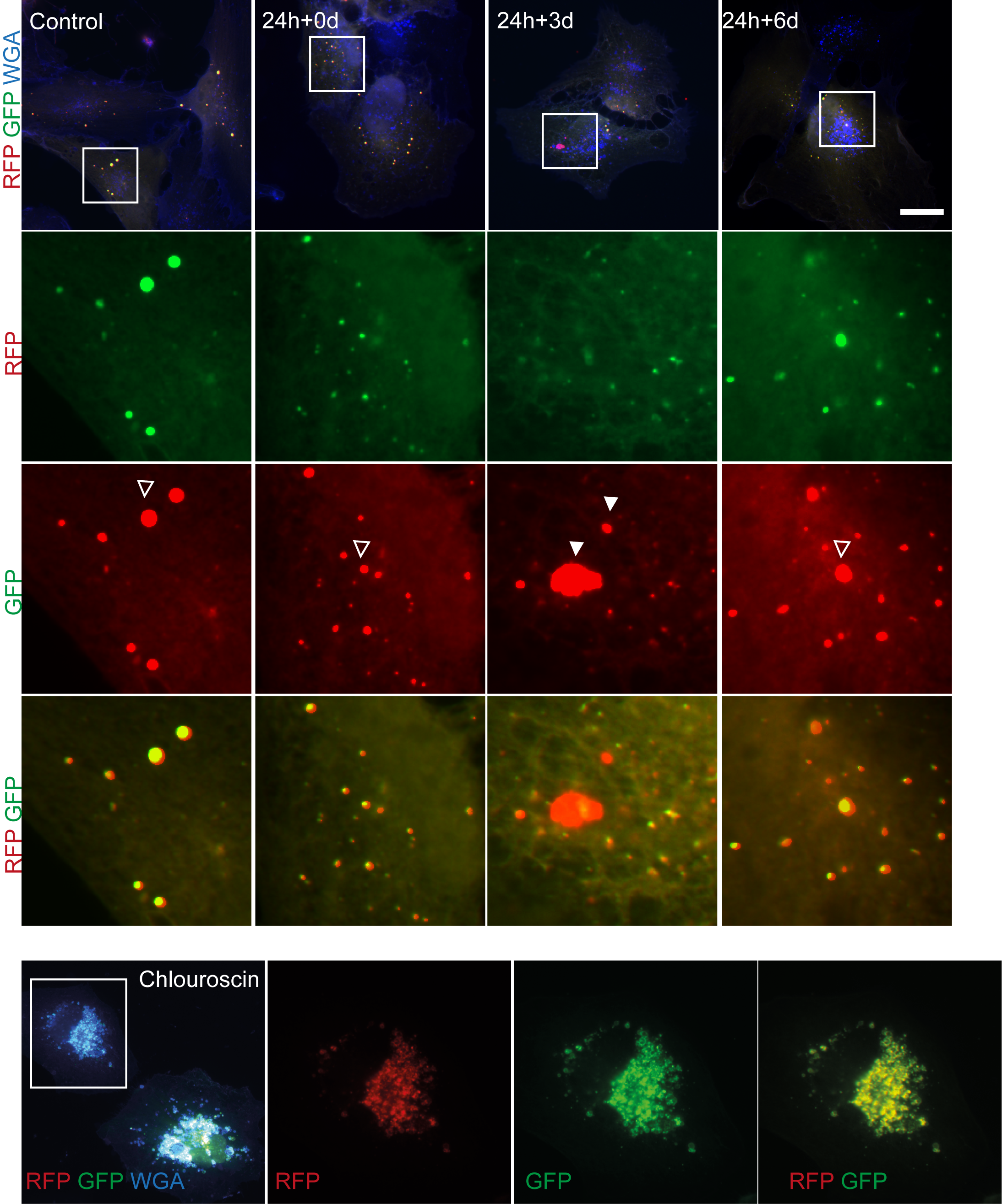

Supplement: Figure 7-1 [file zns999170335so11.tif]

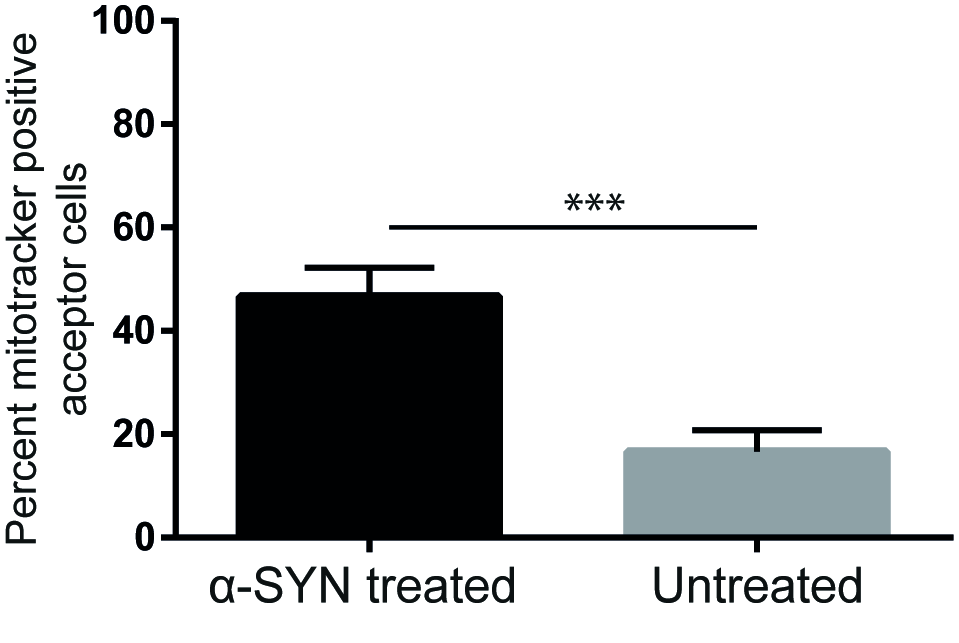

Supplement: Figure 8-1 [file zns999170335so12.tif]
